# Exploring the Impact of Ketodeoxynonulosonic Acid in Host-Pathogen Interactions Using Uptake and Surface Display by Nontypeable Haemophilus influenzae

**DOI:** 10.1128/mBio.03226-20

**Published:** 2021-01-19

**Authors:** Sudeshna Saha, Alison Coady, Aniruddha Sasmal, Kunio Kawanishi, Biswa Choudhury, Hai Yu, Ricardo U. Sorensen, Jaime Inostroza, Ian C. Schoenhofen, Xi Chen, Anja Münster-Kühnel, Chihiro Sato, Ken Kitajima, Sanjay Ram, Victor Nizet, Ajit Varki

**Affiliations:** a Glycobiology Research and Training Center, University of California, San Diego, La Jolla, California, USA; b Center for Academic Research and Training in Anthropogeny, University of California, San Diego, La Jolla, California, USA; c Department of Cellular & Molecular Medicine, University of California, San Diego, La Jolla, California, USA; d Department of Pediatrics, University of California, San Diego, La Jolla, California, USA; e Department of Chemistry, University of California, Davis, California, USA; f Department of Pediatrics, Louisiana State University Health Sciences Center, New Orleans, Louisiana, USA; g Department of Basic Sciences, School of Medicine, Universidad de La Frontera, Temuco, Chile; h Human Health Therapeutics Research Center, National Research Council of Canada, Ottawa, Ontario, Canada; i Clinical Biochemistry, Hannover Medical School, Hannover, Germany; j Bioscience and Biotechnology Center, Nagoya University, Nagoya, Japan; k Division of Infectious Diseases and Immunology, University of Massachusetts Medical School, Worcester, Massachusetts, USA; Washington University School of Medicine; Washington University School of Medicine

**Keywords:** Kdn, nontypeable *Haemophilus influenzae* (NTHi), sialic acid, Neu5Ac, molecular mimicry, antibody, bacterial pathogenesis, CMAH, glycobiology

## Abstract

Surface expression of the common vertebrate sialic acid (Sia) *N*-acetylneuraminic acid (Neu5Ac) by commensal and pathogenic microbes appears structurally to represent “molecular mimicry” of host sialoglycans, facilitating multiple mechanisms of host immune evasion. In contrast, ketodeoxynonulosonic acid (Kdn) is a more ancestral Sia also present in prokaryotic glycoconjugates that are structurally quite distinct from vertebrate sialoglycans. We detected human antibodies against Kdn-terminated glycans, and sialoglycan microarray studies found these anti-Kdn antibodies to be directed against Kdn-sialoglycans structurally similar to those on human cell surface Neu5Ac-sialoglycans. Anti-Kdn-glycan antibodies appear during infancy in a pattern similar to those generated following incorporation of the nonhuman Sia *N*-glycolylneuraminic acid (Neu5Gc) onto the surface of nontypeable Haemophilus influenzae (NTHi), a human commensal and opportunistic pathogen. NTHi grown in the presence of free Kdn took up and incorporated the Sia into its lipooligosaccharide (LOS). Surface display of the Kdn within NTHi LOS blunted several virulence attributes of the pathogen, including Neu5Ac-mediated resistance to complement and whole blood killing, complement C3 deposition, IgM binding, and engagement of Siglec-9. Upper airway administration of Kdn reduced NTHi infection in human-like *Cmah* null (Neu5Gc-deficient) mice that express a Neu5Ac-rich sialome. We propose a mechanism for the induction of anti-Kdn antibodies in humans, suggesting that Kdn could be a natural and/or therapeutic “Trojan horse” that impairs colonization and virulence phenotypes of free Neu5Ac-assimilating human pathogens.

## Abstract (precis)

All cells in vertebrates are coated with a dense array of glycans often capped with sugars called sialic acids. Sialic acids have many functions, including serving as a signal for recognition of “self” cells by the immune system, thereby guiding an appropriate immune response against foreign “nonself” and/or damaged cells.

## INTRODUCTION

Glycan chains on vertebrate cell surfaces and secreted molecules are often capped by nine-carbon backbone monosaccharides called sialic acids (Sias) (1). The unique location, cellular occurrence, and biochemical properties of Sias have imparted important functions in normal and pathophysiology of humans, including interactions with microorganisms, both beneficial and pathogenic (1, 2). Common members of this family include *N*-acetylneuraminic acid (Neu5Ac), *N*-glycolylneuraminic acid (Neu5Gc), and 2-keto-3-deoxy-d-glycero-d-galacto-nonulosonic acid (Kdn). While Neu5Ac and Neu5Gc are prominent in mammals and extensively studied, advancements in Kdn research have been slow and limited since its discovery (3).

Kdn is most abundant in glycoconjugates of cold-blooded vertebrates, and while Sias are generally absent in plants (except for some evidence in green algae) (4, 5), Kdn-glycoconjugates have been reported in microalgae such as Emiliania huxleyi (6) and Prymnesium parvum (7). The most common deuterostome Sia, Neu5Ac, is biosynthesized from condensation of *N-*acetyl-d-mannosamine-6-phosphate (or *N*-acetylmannosamine in prokaryotes) with phosphoenolpyruvate. In an analogous manner, Kdn is formed from mannose-6-phosphate and phosphoenolpyruvate condensation (8, 9). Kdn is resistant to most bacterial and viral sialidases that cleave Neu5Ac from sialoglycoconjugates (10, 11). However, sialidases that can release glycoconjugated Kdn as well as Neu5Ac have been identified in loach liver (10), rainbow trout tissues (11), and bacterium Sphingobacterium multivorum (12, 13). Kdn-specific sialidases lacking activity toward Neu5Ac-glycoconjugates have been reported in Aspergillus fumigatus (14), while the starfish Asterina pectinifera possesses separate Neu5Ac and Kdn sialidases (15). The nine-carbon Kdn is distinct from eight-carbon monosaccharide 3-deoxy-d-manno-oct-2-ulosonic acid (Kdo) (16), which is an integral component of Gram-negative bacterial lipopolysaccharides (17) and present in some algae (18) and cell walls of higher plants (19–21) (see Table S1 in the supplemental material). Moreover, unlike Kdn, Kdo is widely studied (16, 22) including for its role in host-bacterium interaction (23).

10.1128/mBio.03226-20.1 TABLE S1 Comparison between Kdn and Kdo. The table of comparison between Kdn and Kdo is based on references mentioned in the text. Download Table S1, DOCX file, 1.0 MB. © Crown copyright 2021. 2021 Crown This content is distributed under the terms of the Creative Commons Attribution 4.0 International license.

Mammalian cell surfaces are frequently covered with Neu5Ac- and Neu5Gc-terminated glycoconjugates, except in humans, New World monkeys, mustelids (e.g., ferrets), and pinnipeds (e.g., seals), which express only Neu5Ac (24–26) due to independent loss-of-function events involving *CMAH*. *Cmah* encodes cytidine monophosphate (CMP)-Neu5Ac hydroxylase, an enzyme that converts CMP-Neu5Ac to CMP-Neu5Gc, yielding sialoglycomes containing both Neu5Ac and Neu5Gc. Neu5Ac is also present in some bacteria, but prokaryotes do not naturally synthesize Neu5Gc (27, 28). In comparison, Kdn-glycoconjugates have been reported in certain algae (6, 7, 29) and in some bacteria like *Streptomyces* sp. strain MB-8, *Streptomyces* sp. strain VKM Ac-2124 (30, 31), Sinorhizobium fredii SVQ293 (32), and Klebsiella ozaenae serotype K4 (33).

Free Kdn has been reported in human fetal red blood cells (RBCs) (34, 35) and in human malignancies like ovarian, prostate, and throat cancers (35–37). While there is no conclusive evidence for glycosidically bound Kdn-glycoconjugates in normal human tissues, the human Sia synthase enzyme can nevertheless produce both Neu5Ac and Kdn from *N-*acetyl-d-mannosamine-6-phosphate and mannose-6-phosphate, respectively (38). Although the functions of Kdn remain unclear, the conservation of Kdn synthase in vertebrates, including humans, suggests physiological relevance yet to be understood (39–43). Antibodies against the nonhuman Sia, Neu5Gc, have been extensively studied (44, 45), and play important roles in “xenosialitis” associated with different human cancers and pathophysiologies (46, 47). Given the existence of Kdn in humans, we therefore speculate a broader functionality of this Sia, and the antibodies it might generate, than currently known.

Here we sought to expand our understanding of Kdn and explore its role in host-pathogen interactions. We found that humans develop anti-Kdn antibodies early in the first year of life correlating with the appearance of anti-Neu5Gc antibodies. An obligate human upper respiratory tract commensal and opportunistic pathogen, nontypeable Haemophilus influenzae (NTHi), may contribute to anti-Neu5Gc antibody generation in humans (48). Using NTHi as a representative model for any Sia-assimilating bacteria and its Neu5Ac-related pathways as “proof of principle,” we showed free Kdn was taken up and displayed on surface sialoglycans of lipooligosaccharide (LOS), sensitizing the bacterium to killing by complement in normal serum and whole blood. Unlike the protective value of host Neu5Ac incorporation and display, the presence of Kdn on LOS did not disrupt complement C3 or IgM antibody deposition. Finally, we administered Kdn to *Cmah* null (*Cmah^−/−^*) mice expressing a human-like sialoglycan profile to provide evidence that free Sia plays important roles in bacterial Sia uptake and infection *in vivo*.

## RESULTS

### Unlike Neu5Ac, Kdn-containing glycan structures found in prokaryotes do not mimic vertebrate glycoconjugates.

**FIG 1:**
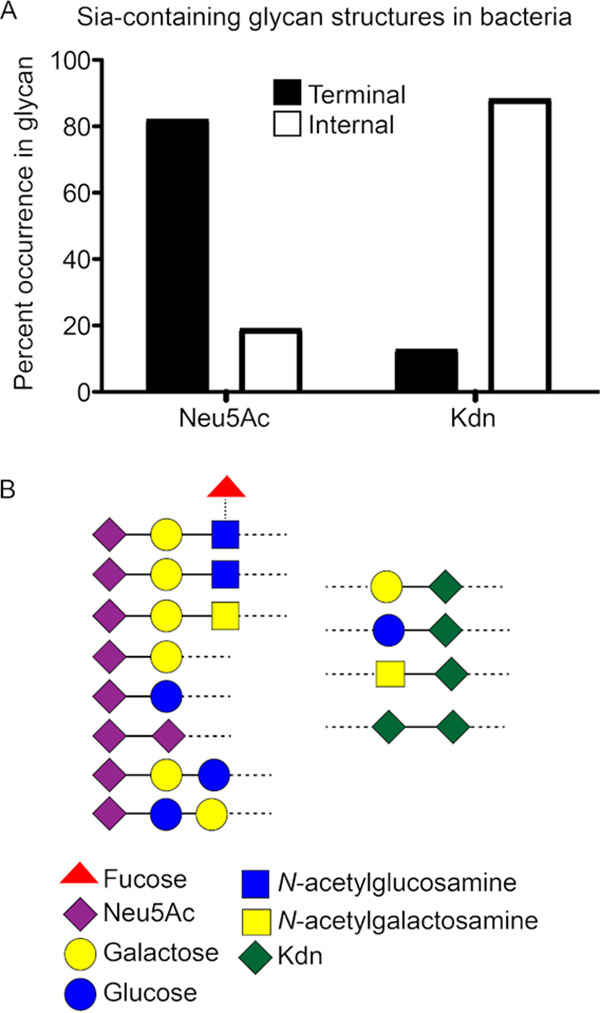
Structural evidence of molecular mimicry in Neu5Ac-containing, but not Kdn-containing, glycans in prokaryotes. (A) The occurrence of Neu5Ac- and Kdn-containing glycan structures in prokaryotes was determined by searching the open access database csdb.glycoscience.ru. The percent quantification is based on data compiled from 432 different Neu5Ac-containing and 55 Kdn-containing prokaryotic structures currently listed in the database. While Neu5Ac is present mainly as the terminal residue on glycan structures, Kdn is mostly an internal residue in the prokaryotic glycans (see Files S1 and S2 in the supplemental material). The black columns represent Sia as the terminal residue in glycan; white columns represent internal residue flanked by glycans. (B) Schematic representation of the common monosaccharides containing Neu5Ac (left) and Kdn (right) epitopes in lipooligosaccharide or capsular polysaccharide as obtained from the database. The pictorial symbols used are in accordance with the Symbol Nomenclature for Graphical Representation of Glycans (SNFG) (135) and are shown in the figure. The dotted line represents glycosidic linkages with the remaining glycoconjugate structure.

While the significance of microbial Neu5Ac-glycan interaction with humans has been extensively studied (49), a comprehensive account and appreciation of prokaryotic sialoglycan structures are lacking. To better understand Sia evolution in prokaryotes, we surveyed available bacterial glycan databases for Neu5Ac- and Kdn-containing glycans (50). Of more than 400 different Neu5Ac-containing glycan structures currently described in bacterial LOS or capsular polysaccharides, about 82% contain Neu5Ac as the terminal monosaccharide, including 16% present as polymers of Neu5Ac residues called polysialic acid (Fig. 1A; see also File S1 in the supplemental material). Sias are the more recently evolved members of a diverse family of nine-carbon monosaccharides called nonulosonic acids (NulOs), with the term “sialic acid” reserved for NulOs present in members of Deuterostome lineages and their associated microorganisms (51). The appearance of Neu5Ac in bacteria may be a result of convergent evolution (52, 53). While genes involved in NulO metabolism are widespread in prokaryotic genomes (51), we observed Neu5Ac largely in bacterial commensals of human microbiome or pathogens involved in human diseases (column B in File S1). Furthermore, the majority of terminal Neu5Ac in bacterial structures is linked to *N*-acetyllactosamine (Fig. 1B), which mimics a common vertebrate sialoglycan structure (54). All these findings are consistent with host molecular mimicry. In contrast, we found Kdn in about 50 different prokaryotic polysaccharide structures, mostly in ubiquitous environmental bacteria, not in the human microbiome or those associated with infectious diseases (column B in File S2). Unlike Neu5Ac, bacterial Kdn is mostly (about 88%) present as internal residue in polysaccharide glycans (Fig. 1A and B; see also Files S1 and S2). These Kdn-containing glycans share few similarities with vertebrate sialoglycoconjugates, although Kdn is sometimes linked to galactose in the sialoglycan (Fig. 1B). Overall, available structural information in glycan databases suggests that Kdn evolution does not fit the paradigm in which prokaryotic Neu5Ac is likely a convergent evolutionary strategy to mimic host sialoglycoconjugates and subvert immune responses (51). Rather, the occurrence of Kdn as a core component of several bacterial polysaccharides indicates a more ancestral Sia, integrated into prokaryotic glycomes and retained only in some eukaryotic taxa.

### Circulating human anti-Kdn antibodies are directed against Kdn-epitopes of vertebrate sialoglycoconjugates.

**FIG 2:**
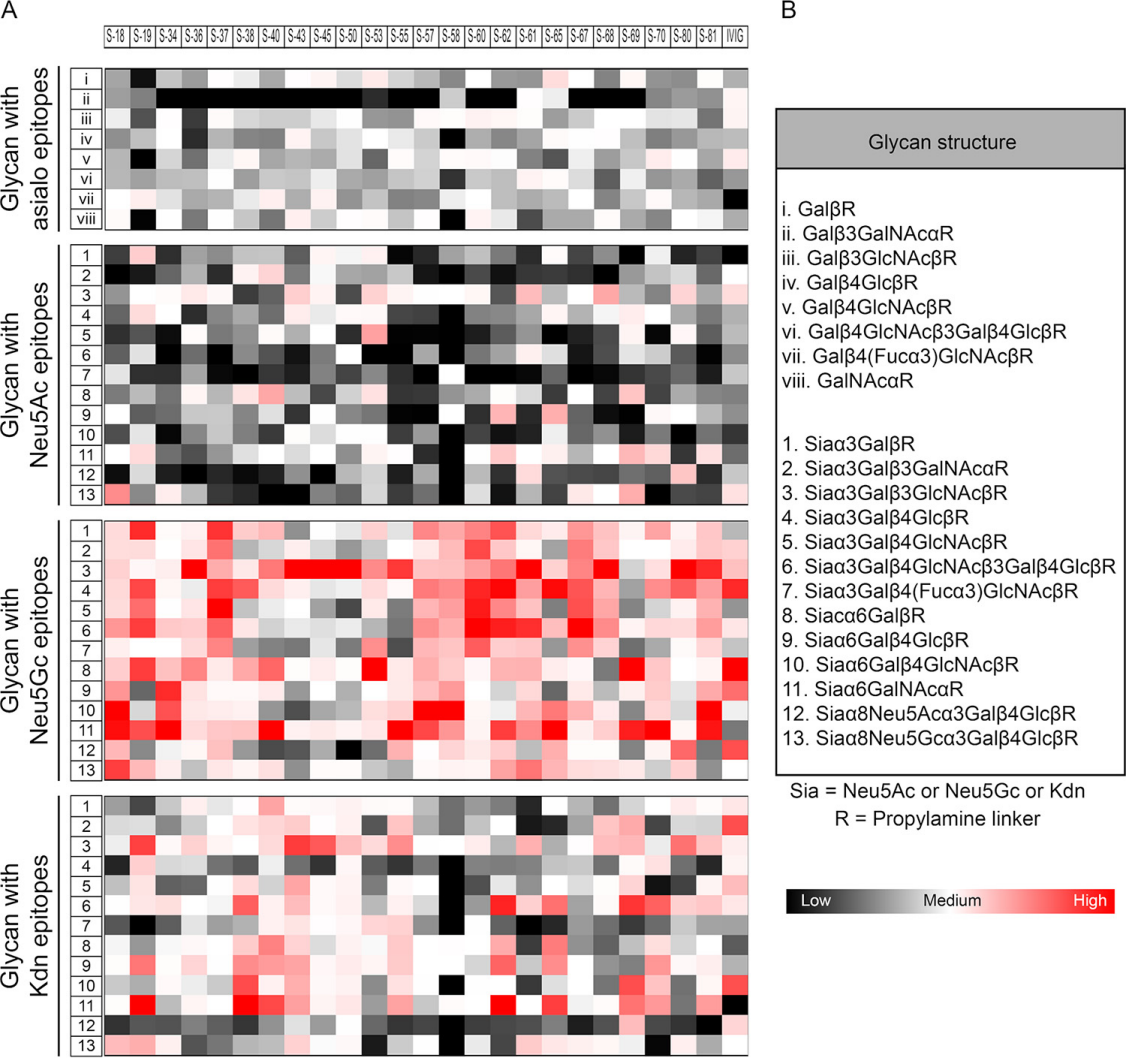
Antigenic specificity of the human anti-Kdn glycan antibodies. Heatmap showing the relative intensity of the fluorophore-conjugated anti-human IgG antibodies binding with the individual glycan on the microarray. Experiment was performed using commercially available pooled human IVIG (last column) as well as individual (*n* = 24) sera. Numbers (S-18 through S-81) on the top represent individual healthy human sera. (A) Each block represents the mean intensity of the antibody binding to the specific asialo- and sialoglycan at four independent spots in the microarray. Glycans are grouped by their nonsialylated (asialo-), Neu5Ac-, Neu5Gc-, and Kdn-terminating structures. The complete list of glycan structures represented is provided in panel B. Each column represents the relative binding preference of IgG antibodies in the corresponding serum toward the specific glycan epitopes. The color code of the heatmap is indicated, and the corresponding glycan in each row matched with the list in panel B.

Human cells are decorated with terminal Neu5Ac and its derivatives, the only Sia class that they can synthesize and incorporate into their sialoglycoconjugates. As a result of the *Cmah*^−^ deletion mutation, humans cannot produce endogenous Neu5Gc (24, 55); however, humans can assimilate dietary Neu5Gc and display it on their glycoconjugates (48). Unlike these two common mammalian Sias, Kdn is normally present in humans only as free monosaccharide, except possibly in some cancers (34, 35). Given the apparent lack of glycosidically linked Kdn, we were intrigued to find the presence of anti-Kdn antibodies in healthy human sera. To probe the corresponding epitopes of human anti-Kdn antibodies, we used an extensive array of diverse glycans displaying Sia as the terminal residue (Fig. 2) (56, 57). These chemoenzymatically synthesized glycans represent oligosaccharide sequences commonly found terminating natural glycoconjugates. Commercially available pooled human intravenous immunoglobulins (IVIG) bound to sialoglycans on the microarray, revealing antibodies against several Kdn-terminating glycans (Fig. 2A, last column). However, the overall anti-Kdn antibody prevalence in IVIG was much lower than those against Neu5Gc-terminated glycans (Fig. 2A). In sera from 24 randomly selected healthy adults (S-18 to S-81), both males and females, various levels of antibodies against Neu5Gc- and Kdn-epitopes were identified with minimal, if any, reactivity against Neu5Ac-glycans (Fig. 2A). To confirm Sia specificity and eliminate cross-reactivity against underlying glycans, we also looked for antibodies against asialoglycan-epitopes representing underlying glycans of the sialoglycan probes (Fig. 2A). Notably, the sialoglycan microarray interactions indicate that antigenic determinants of these human anti-Kdn antibodies are structures (Fig. 2B) similar to mammalian glycoconjugates that normally contain Neu5Ac as the terminal Sia and are present on surfaces of human cells and human-associated microorganisms (54). While the complete antigenic repertoire of anti-Kdn-glycan antibodies is yet to be revealed, our microarray data suggest molecular mimicry of human Neu5Ac-sialoglycoconjugates is involved in the generation of the antibodies (54).

### Human anti-Kdn-glycan antibodies appear during infancy and correlate with the appearance of antibodies against Neu5Gc-glycans.

Several antiglycan antibodies in humans that recognize common epitopes such as galactosyl alpha1-3galactose (αGal) or blood group antigens (anti-ABO group) are thought to develop upon immunization by normal microbiome components (58–60). Antibodies against glycans bearing nonhuman Sia Neu5Gc (48) appear during the first year of life in humans, correlating with dietary introduction of Neu5Gc. However, the introduction of dietary Neu5Gc by itself did not induce antibodies in human-like *Cmah^−/−^* mice (48) suggesting that antigenic triggers of these anti-Neu5Gc antibodies may arise when traces of free dietary Neu5Gc are taken up and metabolically incorporated into surface LOS of human commensals like NTHi (48).

**FIG 3:**
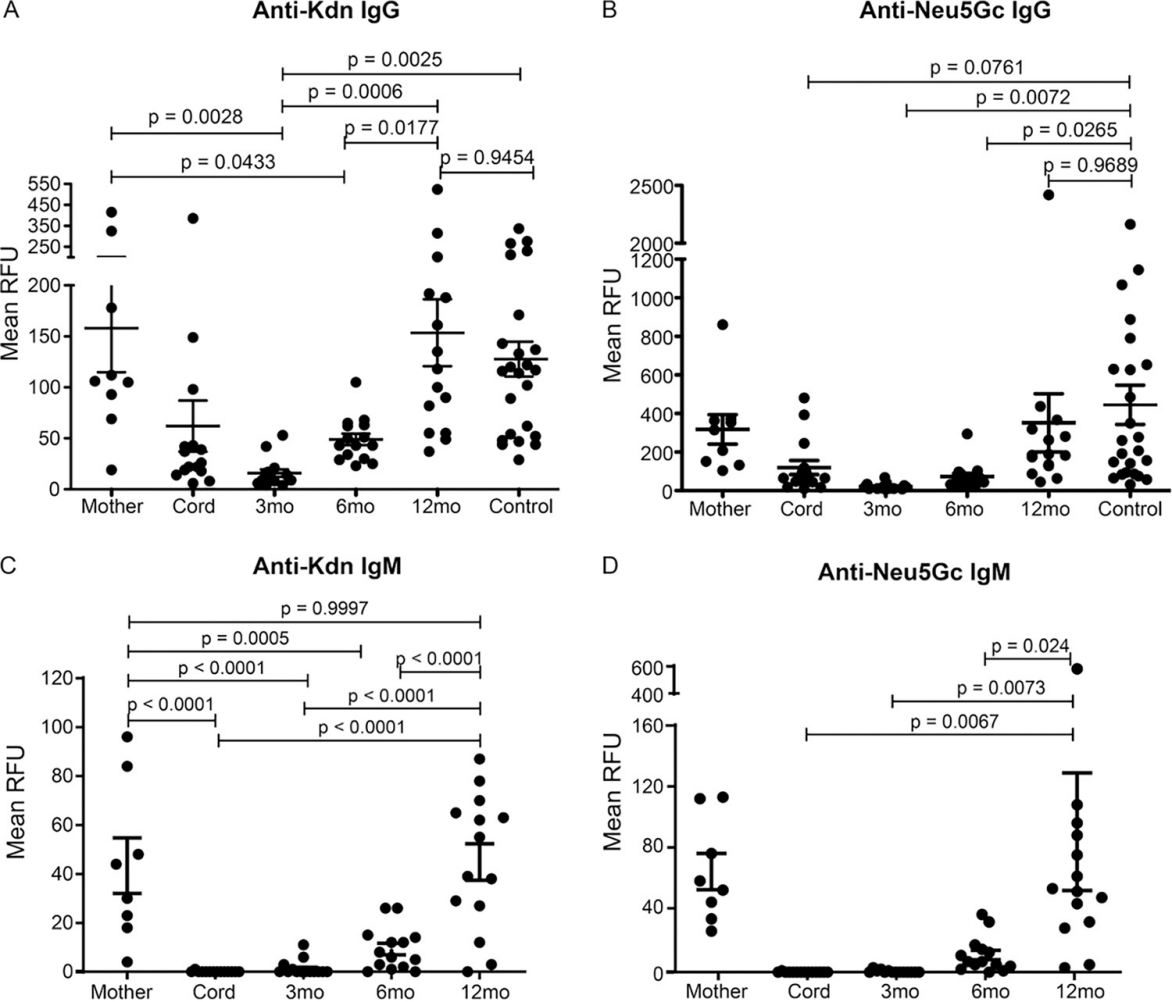
Appearance of anti-Kdn IgG and IgM antibodies in the first year of human life. Binding of human sera to Kdn- and Neu5Gc-containing glycan epitopes was determined by sialoglycan microarray binding assay. Sera were obtained from cord blood (*n* = 15) and the child at 3, 6, and 12 months old and from 9 out of the 15 matched mothers in their third trimester of pregnancy. Control represents sera from blood from healthy human donors (both male and female) (*n* = 24). The average relative fluorescence unit (RFU) in arbitrary units (A.U.) against terminal Kdn (A and C) or Neu5Gc (B and D) containing glycan epitopes with underlying structures similar to vertebrate glycoconjugates are shown. Each dot represents the mean RFU values against all the Kdn (A and C)- or Neu5Gc (B and D)-containing glycans, respectively, for individual serum. (A to D) Relative abundance of the human IgG (A and B) and IgM antibodies (C and D) against the corresponding sialoglycoconjugates. Statistical significance was evaluated using one-way ANOVA with Tukey’s multiple comparison test. The mean ± standard error of the mean (SEM) (error bars) values and the adjusted *P* values are shown in the figure.

In the absence of glycosidically linked Kdn on normal human tissues, we hypothesized that anti-Kdn antibodies might likewise be elicited by the human microbiome. Consistent with this notion, antigenic specificity of anti-Kdn antibodies toward Kdn-glycoconjugates mimicking cell surface glycans in humans (Fig. 2) (54) suggests they could be elicited by some human pathogens and/or commensals that use Neu5Ac in molecular mimicry of the host. In this model, anti-Kdn antibodies would appear following postnatal seeding of newborns with the microbiome from their mother and/or the environment (61–64). To determine when antibodies against Kdn-epitopes first appear, we performed sialoglycan microarray binding assays with a previously reported collection of sera (*n* = 15) obtained at birth and at 3, 6, and 12 months of age (48). We also investigated sialoglycan binding of sera from nine out of 15 matched mothers and healthy adults (*n* = 24, control) (Fig. 3). Sera were tested for interaction with sialoglycoconjugates presenting terminal Kdn in different α-linkages with underlying glycans as in Fig. 2. We observed IgG antibodies against Kdn-glycans in cord blood, but their abundance dropped at 3 months of age, suggesting that they arose from transplacental transfer of maternal antibodies during pregnancy (65). Anti-Kdn IgG antibodies reappeared by 6 months of age and within the first year of life attain levels similar to those in adults (Fig. 3A). The dynamics of anti-Kdn antibodies are similar to previously reported antibodies against the Neu5Gcα2–6Lacβ-epitope, appearing during the first year (48) and coinciding with the course of microbiome establishment in infants (66). Here we expanded on that observation to show that anti-Neu5Gc IgG antibodies against diverse Neu5Gc-epitopes are generated in this time window (Fig. 3B). We also found that while the kinetics of anti-Kdn IgG antibody appearance are similar to those of anti-Neu5Gc antibodies, the relative serum abundance of the former is much lower, perhaps reflecting reduced availability or antigenicity of natural Kdn-epitopes compared to Neu5Gc-epitopes in humans.

To ascertain whether anti-Kdn-glycan antibodies are generated postnatally following antigen presentation or are “natural” antibodies encoded in germ line (67), we further looked for IgM in the infant and adult sera. Unlike IgG, maternal IgM antibodies fail to cross the placenta, and the presence of IgM antibody in cord blood would suggest they were generated by the newborn prior to any antigen exposure. Anti-Kdn-glycan IgM antibodies did not appear in infant blood until 6 months of age and gradually increased thereafter (Fig. 3C). IgM antibodies against Kdn-glycans were also present in mothers suggesting their persistence in adults. By 12 months, these antibodies reached maternal levels corresponding to establishment of the normal microbiome in different niches throughout human body (63, 64). Appearance of IgM antibodies against Neu5Gc-glycoconjugates showed similar profiles (Fig. 3D). Thus, antibodies against Kdn-epitopes are not germ line-encoded “natural” antibodies (68) but instead are generated by the adaptive immune system of the newborn following antigen exposure.

### Free exogenous Kdn can be incorporated into NTHi surface LOS.

**FIG 4:**
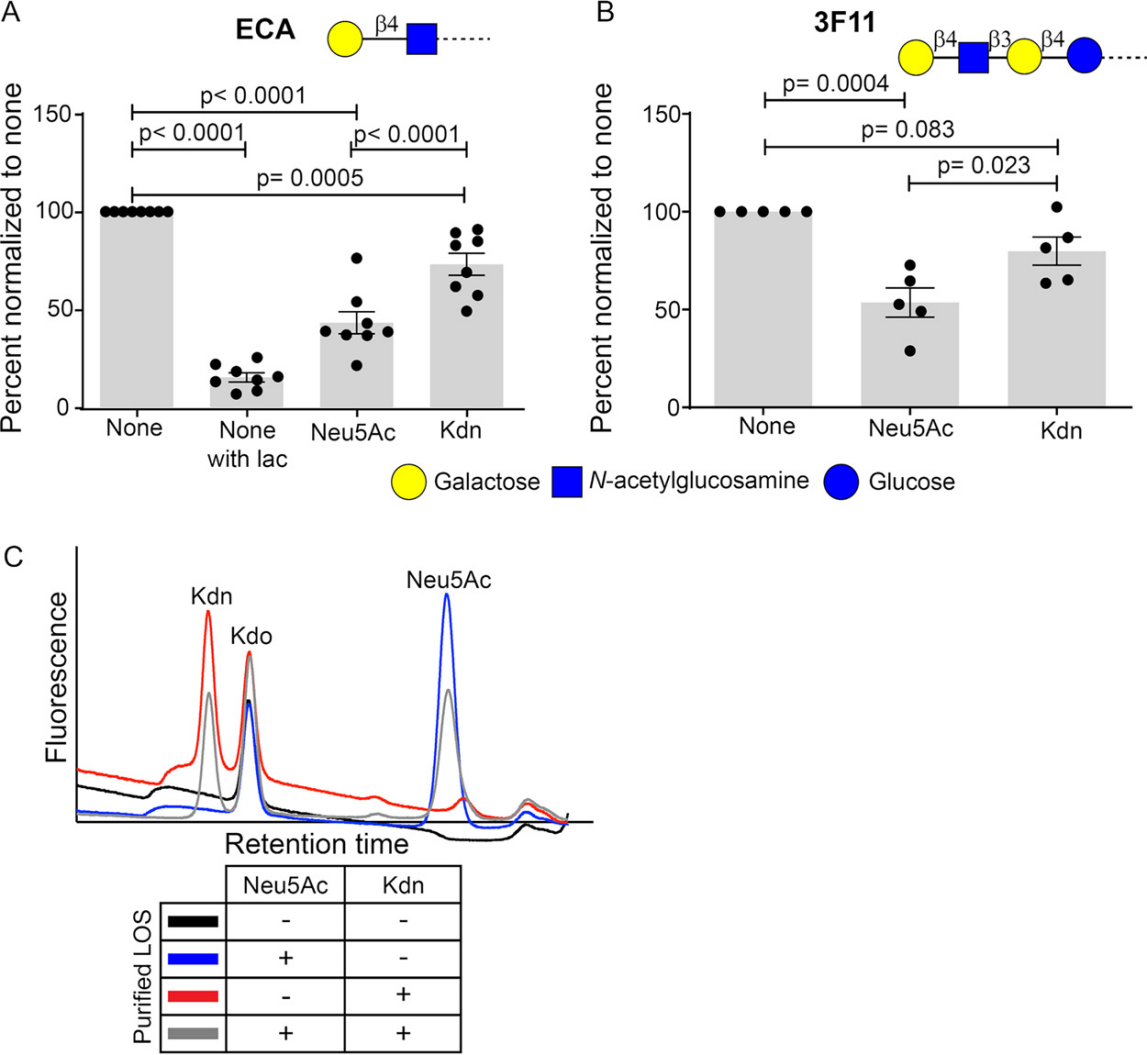
Exogenous Kdn is taken up and displayed by NTHi on its LOS. Surface sialylation of NTHi 2019 grown in the presence or absence of Neu5Ac or Kdn was determined using plant lectin, ECA (A) and mouse monoclonal IgM antibody 3F11 (B) in a flow cytometry-based assay. The bacteria were incubated with biotinylated lectin at 37°C or with mouse IgM antibody (Ab) 3F11 at room temperature (RT) for 30 min and then probed with fluorophore-conjugated secondary antibodies for analysis via flow cytometry. The binding epitopes of each of the reagents are shown within the panel using the symbols in accordance with SNFG nomenclature. “None” indicates the bacteria grown in the absence of Sia, while “Neu5Ac” or “Kdn” indicates the presence of the corresponding Sia in growth media. ECA binding with unsialylated bacteria done in the presence of 100 μM free lactose (none with lac) was used as a control to show the inhibition of ECA binding, since lactose is a competitive inhibitor of the lectin. Each dot represents the mean fluorescence intensity of an independent biological experiment (*n* = 8 for ECA; *n* = 5 for 3F11). Fluorescence values were expressed relative to that seen with the unsialylated samples in the same experiment to normalize for day-to-day variation. Statistical significance was determined using one-way ANOVA with Tukey’s multiple comparison test. Bars represent the mean (SEM) for each assay (A and B), and all the adjusted *P* values are shown in the figure. (C) Bacterial LOS was purified by hot phenol-water extraction from NTHi 2019 grown in the presence or absence of one or both Sias. Representative profiles of HPLC analysis following acid hydrolysis and DMB derivatization of purified LOS are shown. The fluorescent intensity peaks of the individual α-keto acid are identified in the figure. Kdo represents 3-deoxy-d-manno-oct-2-ulosonic acid, a key component of Gram-negative bacterial LOS and serves as the internal control in each of the LOS preparations. The table below the graph shows the bacterial cultures used to purify the corresponding LOS whose profile is shown. The presence (+) or absence (-) of the Sia in growth media of the bacteria is indicated.

A contributing factor to the development of antibodies against nonhuman Sia Neu5Gc is proposed to be presentation of glycosidically linked Neu5Gc on surface LOS of uniquely human bacteria NTHi (48). Appearance of anti-Kdn antibodies shows a profile similar to the occurrence of antibodies against NTHi surface oligosaccharides (48) and coincides with the onset of NTHi colonization in the newborn (61, 66, 69). To test our hypothesis that the natural microbiome may contribute to generation of anti-Kdn antibodies, we utilized NTHi as a model exogenous Sia-assimilating microbe. Clinical isolate NTHi 2019 (70) was grown in the presence of 100 µM free Sias, and Kdn incorporation onto bacterial LOS was measured. NTHi 2019 takes up environmental free Neu5Ac, and the resulting major sialylated surface glycan is Neu5Acα2-3Galβ1-4GlcNAc (23, 71). To determine whether free Kdn is similarly displayed, we used the plant lectin Erythrina cristagalli lectin (ECA) (72) that recognizes Galβ1-4GlcNAc termini when they are not capped with Sia, as confirmed by our sialoglycan microarray (see Fig. S1 in the supplemental material) and further ensured by a strong reduction of ECA binding in the presence of inhibitory concentrations of the competitive sugar lactose (Fig. 4A, Fig. S2A). As expected, ECA bound NTHi 2019 when grown in Sia-free media, and the binding was reduced after feeding and incorporation of Neu5Ac or Kdn (Fig. 4A, Fig. S2A), indicating glycosidically linked Sia. Mouse monoclonal antibody (mAb) 3F11 specifically binds terminal lactosamine of unsialylated Galβ1-4GlcNAcβ1-3Galβ1-4Glc and is widely used to demonstrate the surface sialylation of different bacteria, including NTHi 2019 (23). Although the cognate disaccharide epitope is the same as ECA, mAb 3F11 is an IgM antibody requiring multivalent binding. As with ECA, we observed increased binding of 3F11 to NTHi 2019 grown in the absence of Sia, suggesting a reduction in its unsialylated epitopes upon sialylation (Fig. 4B, Fig. S2B). These results suggest that like Neu5Ac, Kdn is displayed on NTHi 2019 surface with an underlying lactosamine structure.

10.1128/mBio.03226-20.2 FIG S1 Sialoglycan microarray binding of plant lectins. Biotinylated plant lectins were allowed to interact with sialoglycan microarray containing different Neu5Ac- or Kdn-terminating as well as some asialo-glycans as indicated in the left side of the panels. RFU indicate relative fluorescence unit. Erythrina cristagalli lectin (ECA), Maackia amurensis lectin I (MAL I). The color code of the right-hand column is indicated below the figure. Download FIG S1, TIF file, 1.4 MB. © Crown copyright 2021. 2021 Crown This content is distributed under the terms of the Creative Commons Attribution 4.0 International license.

10.1128/mBio.03226-20.3 FIG S2 Lectin staining of wild-type NTHi 2019 and mutants in presence of Kdn. Representative flow cytometry profiles of wild-type (WT) bacteria grown in the presence of Neu5Ac or Kdn, followed by staining with plant lectin, ECA (A) or mouse monoclonal antibody, 3F11 (B). Flow cytometry profiles of wild-type NTHi 2019 and lyase-depleted mutant (Δ*nanA*) (C) following ECA binding. Figure legends show the media condition used for bacterial growth prior to the assay. “None” indicates media without any Sia. “Neu5Ac” or “Kdn” indicates the presence of the corresponding Sia in growth media. “None, Lac” shows bacteria grown without any Sia and where ECA binding was done in the presence of free lactose, a competitive inhibitor of ECA. Ab contl indicates fluorescent secondary antibody control for 3F11 staining. Download FIG S2, TIF file, 2.1 MB. © Crown copyright 2021. 2021 Crown This content is distributed under the terms of the Creative Commons Attribution 4.0 International license.

Neu5Ac is an important component of NTHi LOS (23, 73). To confirm that free exogenous Kdn is incorporated into NTHi LOS, we purified LOS from bacteria grown in the presence of Sia using hot phenol-water extraction (74). Purified LOS was then treated to release glycosidically bound Sias for derivatization using 1,2-diamino-4,5-methylenedioxybenzene dihydrochloride (DMB) and fluorescence detection in high performance liquid chromatography (HPLC) (75, 76). Peaks eluting at the same retention time as Neu5Ac or Kdn were observed in LOS purified from NTHi grown in the corresponding Sia (Fig. 4C). To determine whether free Kdn can be incorporated in the presence of Neu5Ac, we grew NTHi 2019 with both Neu5Ac and Kdn (1:10 molar ratio). Analysis of the purified LOS indeed showed fluorescence peaks corresponding to retention times of both Sias (Fig. 4C). A fluorescence peak corresponding to retention time of Kdo, a key component of Gram-negative bacterial LOS, was seen in all samples confirming LOS purification. Altogether, the data indicate that exogenous Kdn can be taken up and incorporated in NTHi 2019 LOS in a manner similar to free Neu5Ac. Notably, when grown without Neu5Ac, NTHi 2019 incorporates endogenously synthesized Kdo to the terminal lactosamine on its LOS. However, the addition of exogenous Neu5Ac to growth media is associated with the replacement of this terminal Kdo by Neu5Ac (23).

### LOS incorporation of exogenous Kdn in diverse NTHi strains.

**FIG 5:**
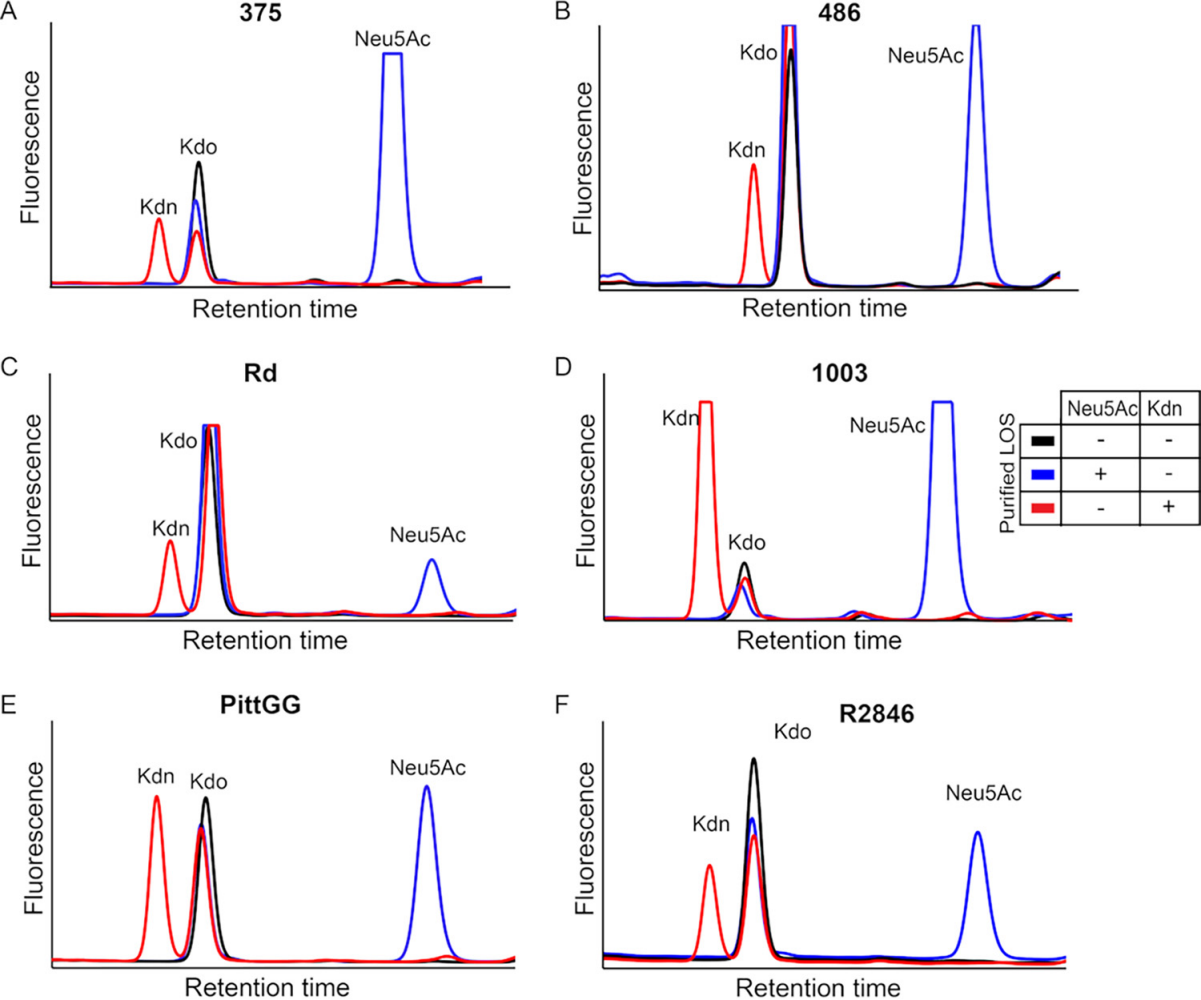
Diverse NTHi strains incorporate exogenously provided Kdn. Six NTHi strains were grown in the presence of either Neu5Ac or Kdn and the bacterial LOS was purified by hot phenol-water extraction. HPLC analysis of acid-hydrolyzed and DMB-derivatized LOS shows fluorescent intensity peaks corresponding to Neu5Ac or Kdn in NTHi 375 (A), 486 (B), Rd (C), 1003 (D), PittGG (E) and R2846 (F). The individual Sia and Kdo as the internal control of the LOS preparations are identified as in Fig. 4C. The table shows the growth condition of bacterial cultures used to purify the LOS with (+) or without (−) the corresponding Sia added.

Structural heterogeneity of NTHi LOS prompted us to determine whether the exogenous Kdn incorporation is strain specific or can occur in others beyond NTHi 2019. We examined six different NTHi strains, including some with well-characterized LOS structures, namely, strains 375, 486, Rd, 1003, PittGG, and R2846 (77–81). HPLC analysis of purified LOS from the bacteria, grown either in Neu5Ac or Kdn, showed the presence of the corresponding glycosidically bound Sia on the LOS (Fig. 5). These data indicate the ability of various NTHi strains to take up and assimilate Kdn.

### NTHi uses the same machinery to take up and degrade exogenous Kdn as for Neu5Ac.

**FIG 6:**
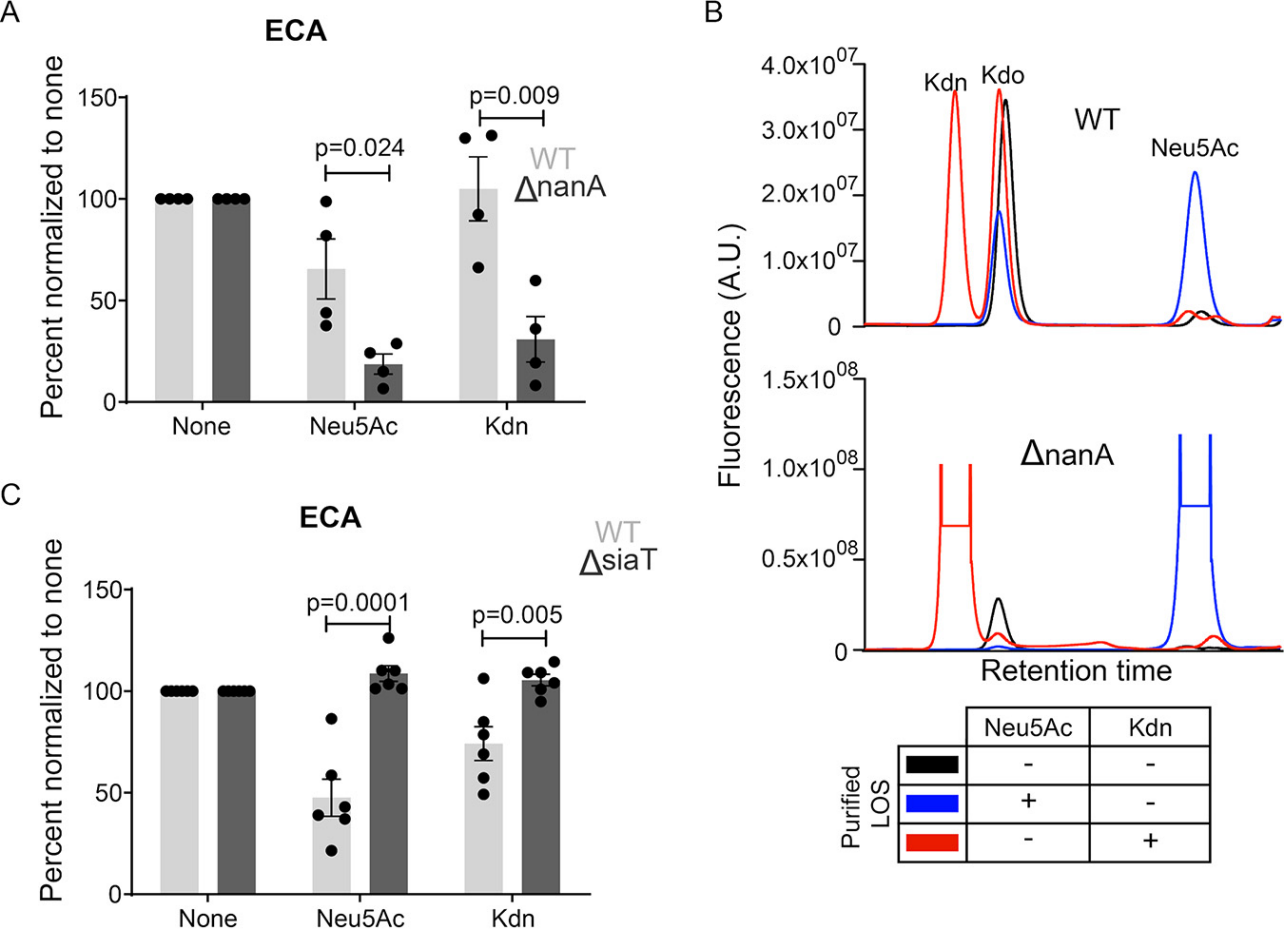
NTHi utilizes the same machineries for Kdn uptake and degradation as those for Neu5Ac. (A) Presence of Sia on the surface of wild-type (WT) NTHi 2019 and lyase deletion mutant 2019Δ*nanA* was determined using ECA binding. The bacterial treatment and data analysis are the same as described in the legend to Fig. 4. (B) HPLC profiles of LOS purified from NTHi 2019 or 2019Δ*nanA* grown in the presence or absence of 100 µM Neu5Ac or Kdn. The relative fluorophore intensity of the α-keto acid in arbitrary units (A.U.) present in the corresponding LOS is indicated on the *y* axis. Kdo represents an internal control as in Fig. 4C. The table below the graph shows the bacterial culture condition used to purify the corresponding LOS. (C) ECA binding assay with NTHi 2019 and the transporter deletion mutant 2019Δ*siaT* grown in the presence or absence of Neu5Ac or Kdn. The dots are the same as described in the legend to Fig. 4. Statistical significance was determined using Student *t* test. All the values were normalized to the values for the unsialylated samples for the individual experiment to avoid day-to-day variation. Mean with SEM from independent experiments (*n* = 4 for panel A; *n* = 6 for panel C) was calculated. “None” indicates the bacteria grown in the absence of Sia, while “Neu5Ac” or “Kdn” indicates the presence of the corresponding Sia in growth media. Adjusted *P* values are shown.

In addition to capping surface LOS, NTHi can utilize Neu5Ac as a carbon source (71, 82, 83). Following the uptake, free Neu5Ac is degraded by bacterial Sia lyase into *N*-acetylmannosamine (ManNAc) and pyruvate. ManNAc is then converted to intermediates like glucosamine-6-phosphate and enters metabolic pathways (83). To determine whether Kdn is metabolized in a similar manner, we first examined growth of NTHi 2019 in the presence of Kdn (Fig. S3). The bacteria were grown in glucose-augmented, RPMI-based media freshly supplemented with NAD and protoporphyrin IX—two essential components for NTHi growth (84). Bacterial growth in Kdn-containing media was comparable to that in Neu5Ac. However, in the presence of its preferred carbon source (i.e., glucose) in basal media, no significant differences in overall growth were observed regardless of Sia presence. Hence, we sought to specifically determine whether Kdn can be utilized by NTHi and whether it is catabolized using a similar machinery as for Neu5Ac. For NTHi 2019, the Neu5Ac lyase has been identified as NanA (85). To elucidate whether NanA participates in Kdn catabolism, we employed 2019Δ*nanA* mutant (85) that lacks the lyase and cannot degrade Neu5Ac upon its uptake. As a result of more available intact Sia, the 2019Δ*nanA* mutant strain displays increased LOS sialylation compared to the wild-type (WT) parent strain, where Neu5Ac is utilized for both surface sialylation and catabolism (85). Compared to WT, the lyase-deficient mutant showed increased surface capping by both Neu5Ac and Kdn, as indicated by the more pronounced reduction in ECA binding (Fig. 6A, Fig. S2C). Furthermore, purified LOS from the 2019Δ*nanA* mutant showed greater Kdn incorporation than the WT (Fig. 6B) when grown under similar conditions with equimolar Sia concentrations.

10.1128/mBio.03226-20.4 FIG S3 Growth of NTHi 2019 in presence of Kdn. Following overnight growth in chocolate II agar, log phase starter culture was diluted, and growth curve was determined in the presence of 100 µM Sia (*n* = 2). “None” indicated growth without any Sia, “Neu5Ac” or “Kdn” indicates the presence of the corresponding Sia in growth media during the assay. Mean with SEM is shown. Download FIG S3, TIF file, 1.7 MB. © Crown copyright 2021. 2021 Crown This content is distributed under the terms of the Creative Commons Attribution 4.0 International license.

Although Neu5Ac is important for NTHi commensalism and pathogenesis, the bacterium solely relies on exogenous free Neu5Ac, utilizing a tripartite ATP-independent periplasmic transporter to sequester the Sia from its environment (85). For NTHi 2019, components of this transporter are integral membrane protein SiaT (also known as SiaQM) and SiaP, the membrane receptor for the ligand (i.e., Sia) binding (86, 87). Compared to the WT, the previously reported transporter-deficient mutant 2019Δ*siaT* (85) remained uncapped when grown in the presence of Sia and did not show reduced ECA binding (Fig. 6C, Fig. S4A), suggesting that ECA-epitopes have not been abolished by Sia capping. Furthermore, no increase in Maackia amurensis lectin I (MAL I) (a plant lectin recognizing Sia, either Neu5Ac or Kdn, α2-3-linked to underlying glycan) binding of 2019Δ*siaT* mutant was seen compared to unsialylated bacteria upon growth in Neu5Ac or Kdn, suggesting the absence of sialylated MAL I-epitopes (Fig. S4B and S4C). Together, these data show that NTHi 2019 utilizes the same enzymes (and likely the same pathway) for the uptake and metabolism of Kdn as it does for Neu5Ac.

10.1128/mBio.03226-20.5 FIG S4 Interaction of wild-type NTHi 2019 and transporter-depleted mutant with plant lectins. Wild-type NTHi 2019 (WT) and Δ*siaT* mutant were grown in the presence of Neu5Ac or Kdn, and the surface sialoglycoconjugates of the bacteria were determined using plant lectins, ECA and MAL I. The representative flow cytometry profiles of bacteria following binding with ECA (A) and MAL I (B). Quantification of MAL I interaction with WT and mutant NTHi 2019 from two experiments (C). The binding epitope is shown using the symbols in accordance with SNFG. Each dot in the graph represents the mean value of one independent experiment. Mean with SEM is shown in the figure. Figure legends show the media condition used to grow the bacterial culture prior to the assay. “None” indicates the bacteria grown in the absence of Sia, “Neu5Ac” or “Kdn” indicates the presence of the corresponding Sia in growth media. Ab contl. indicates fluorescent antibody control. Download FIG S4, TIF file, 2.6 MB. © Crown copyright 2021. 2021 Crown This content is distributed under the terms of the Creative Commons Attribution 4.0 International license.

### Unlike Neu5Ac, Kdn does not protect against serum and whole blood killing.

**FIG 7:**
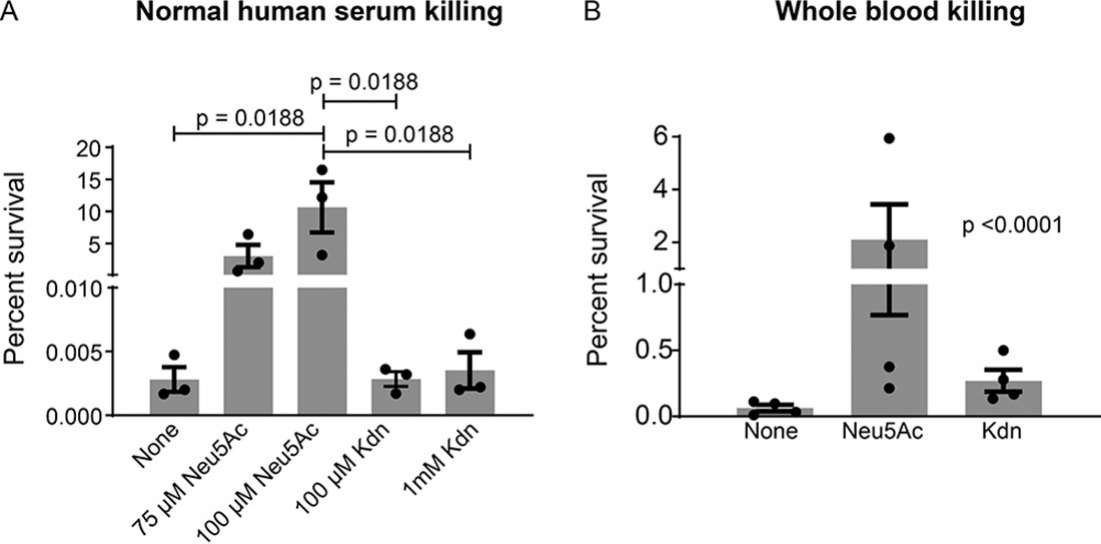
Unlike Neu5Ac, Kdn incorporation does not provide protection against serum and whole blood killing. (A) NTHi 2019 was grown with or without Neu5Ac or Kdn and incubated in the presence of 10% pooled normal human sera at 37°C for 30 min. Percent survival was determined from CFU counts of bacteria recovered following serum incubation relative to the inoculum. The amounts of Sia added during bacterial growth are shown on the *x* axis. (B) Bactericidal effect of hirudin-anticoagulated whole blood collected from healthy human donors is shown (*n* = 4). Each dot in the figures represents the corresponding value in each experiment. Mean with SEM (*n* = 3 for serum killing; *n* = 4 for whole blood killing) and all the adjusted *P* values are shown. Statistical significance was determined using one-way ANOVA with Tukey’s multiple comparison test (A) and Bartlett’s test (B).

Incorporation of Neu5Ac on LOS allows NTHi to resist complement-mediated serum killing (88–91). Since Kdn can be incorporated into LOS, we asked whether its presence affected NTHi survival in human sera. Sialylated NTHi 2019 was incubated with normal human sera, and its survival was assessed. While NTHi supplemented with Neu5Ac survived serum killing, NTHi bacteria grown in Kdn were killed as readily as unsialylated bacteria (Fig. 7A). To eliminate the possibility of inefficient sialylation with Kdn (71), we also grew the bacteria with a lower concentration (75 µM) of Neu5Ac and a higher concentration (1 mM) of Kdn. The bacteria grown in the presence of 75 µM Neu5Ac demonstrated 1,000-fold better survival compared to those grown in 1 mM Kdn (Fig. 7A). Of note, growth of bacteria in the presence of both Kdn and Neu5Ac showed that the effects of Neu5Ac (i.e., serum resistance) predominated (Fig. S5A). Sia modification of the LOS has also been associated with bloodstream survival of invasive NTHi (92). In survival assays with hirudin-anticoagulated human whole blood (93, 94), NTHi grown in the presence of Kdn showed decreased survival compared to those grown in Neu5Ac (Fig. 7B).

10.1128/mBio.03226-20.6 FIG S5 Bacterial interaction with human immune system when grown in the presence of Kdn. (A) Bacteria grown in the presence of different concentrations of Neu5Ac, Kdn, or both were incubated with 10% pooled normal human sera for 30 min at 37°C. Percent survival was determined by the CFU counts of the bacteria at 30 min relative to 0 min. Mean with SEM is shown. NTHi 2019 grown in the presence of Neu5Ac or Kdn, was incubated in the presence (+NHS) or absence (-NHS) of pooled human sera for 5 min to determine IgG or IgM antibodies or complement C3c deposition. IgG and IgM deposition were tested using heat-inactivated normal human sera. For factor H 6/7 binding (FH6/7), the bacteria were incubated with purified FH6/7 supernatant. The representative flow cytometry profiles of IgG antibody (B), IgM antibody (D), complement FH6/7 (E), and C3c (G) deposition are shown. Figure legends within the panel indicate the media used for bacterial growth prior to each assay. “None” means absence of Sia. “Neu5Ac” or “Kdn” indicates the presence of corresponding Sia in the media. Quantification of IgG (*n* = 4) and FH6/7 (*n* = 3) deposition assay is shown in panels C and F, respectively. Each dot represents the mean fluorescence intensity of an independent experiment. Mean with SEM is shown. Ab contl. indicates fluorescent antibody control. Download FIG S5, TIF file, 2.4 MB. © Crown copyright 2021. 2021 Crown This content is distributed under the terms of the Creative Commons Attribution 4.0 International license.

10.1128/mBio.03226-20.7 FILE S1 Prokaryotic Neu5Ac-containing sialoglycoconjugates. The survey of the Neu5Ac-containing prokaryotic sialoglycan structures from bacterial carbohydrate structure database is presented here. The name of the organism (column A) containing the corresponding glycan structure (columns D and E) is indicated. The nature of association of the bacteria in column A with human is indicated (column B). Compound ID (column C) is the identity of the structure in the database. Graphical representation of the sialoglycan structures using the symbols in accordance with SNFG nomenclature is shown (column E). Download FILE S1, XLSX file, 1 MB. © Crown copyright 2021. 2021 Crown This content is distributed under the terms of the Creative Commons Attribution 4.0 International license.

10.1128/mBio.03226-20.8 FILE S2 Prokaryotic Kdn-containing sialoglycoconjugates. The survey of the Kdn-containing prokaryotic sialoglycan structures from bacterial carbohydrate structure database is presented here. Details of the columns are the same as in File S1. Download FILE S2, XLSX file, 0.1 MB. © Crown copyright 2021. 2021 Crown This content is distributed under the terms of the Creative Commons Attribution 4.0 International license.

**FIG 8:**
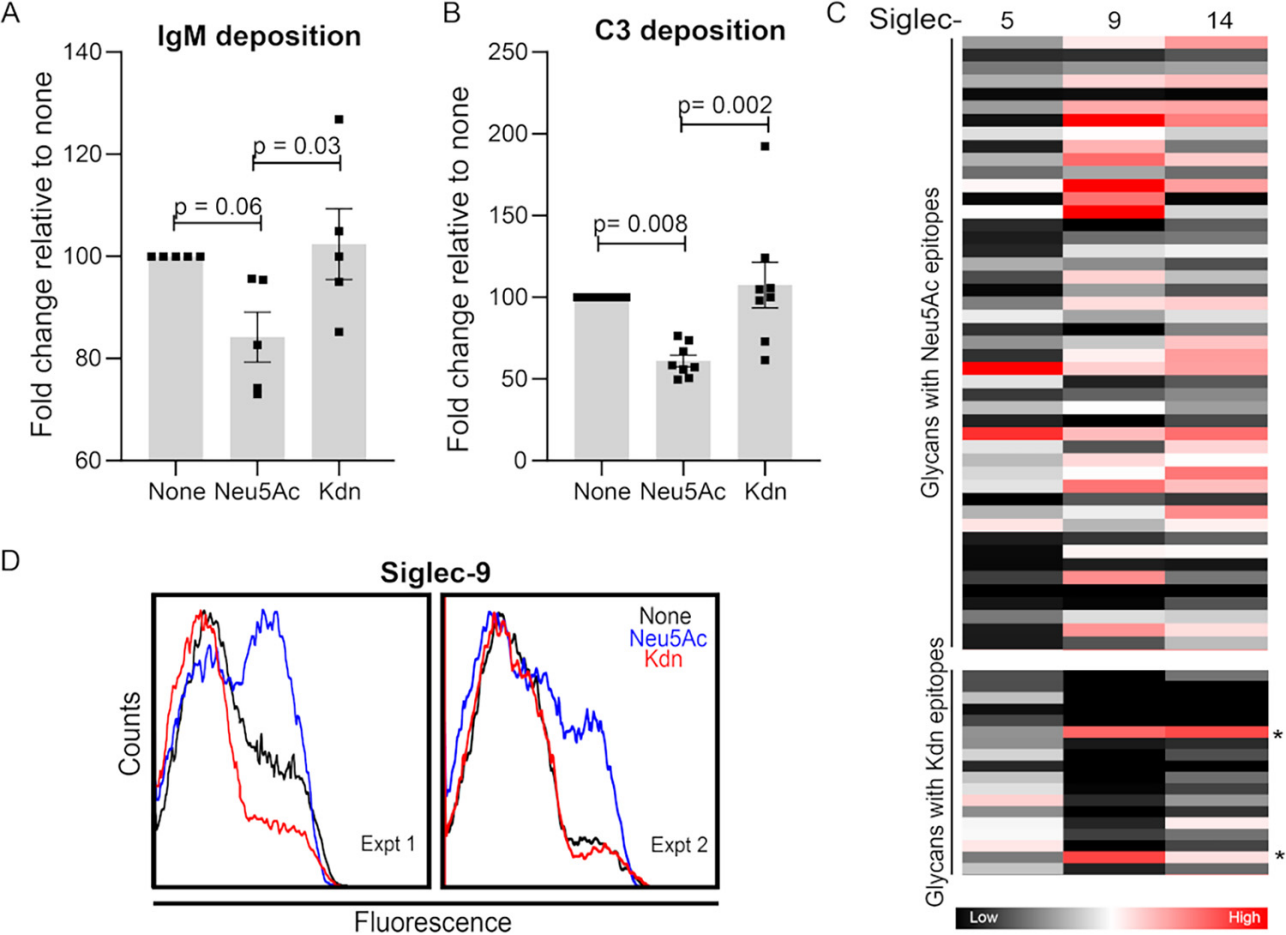
Incorporated Kdn does not abolish complement C3 and IgM antibody deposition and does not substitute for Neu5Ac in interaction with Siglecs. Bacteria grown in the presence of Neu5Ac or Kdn were incubated with 10% pooled normal human sera for 5 min at 37°C and IgM antibody (A) and complement factor C3 (B) deposition were determined using the corresponding antibody staining in flow cytometry. For IgM deposition, the bacteria were incubated with heat-inactivated sera. Each dot represents an independent experiment (*n* = 5 for panel A and *n* = 8 for panel B). The data were normalized to the values for unsialylated bacteria to eliminate the daily variation. Statistical significance was determined using one-way ANOVA with Tukey’s multiple comparison test. Mean with SEM and adjusted *P* values are shown in the figure. (C) Heatmap showing the binding of Neu5Ac- and Kdn-containing glycan epitopes to purified recombinant Fc chimeric proteins of human Siglec-5, -9, and -14. The map represents the binding affinities of each protein toward the corresponding glycans determined from the glycan array binding experiment. The complete list of the glycan used are shown in File S3 in the supplemental material. Glycans with lactose underlying structure are indicated by an asterisk. Each block corresponds to a distinct glycan with terminal Sia as indicated in the left. The color of each block represents the mean binding intensity done in technical quadruplet. Color code is indicated below. (D) Representative flow cytometry profiles showing Siglec-9 binding with NTHi 2019 grown in the presence or absence of Neu5Ac or Kdn in two independent biological experiments (experiments 1 and 2). Figure legend shows the Sia added to the bacterial growth media. “None” indicates the bacteria grown in the absence of Sia, while “Neu5Ac” or “Kdn” indicates the presence of the corresponding Sia in growth media.

10.1128/mBio.03226-20.9 FILE S3 Complete list of the glycans used in the sialoglycan microarray for Siglecs. The detailed structure of the sialoglycans used to determine the binding of purified Siglec-5, -9, and -14 is shown. The binding intensity towards the corresponding glycan in shown in the heatmap in column B, C, and D, respectively (same heatmap as in Fig. 8C). The red indicates highest binding and black indicates the least binding. R, propylamine linker present in the underlying glycan structure; Gal, galactose; GalNAc, *N*-acetylgalactosamine; Glc, glucose; GlcNAc, *N*-acetylglucosamine; Fuc, l-fucose. The linkage between the monosaccharides is indicated as α- or β- with numbers. Download FILE S3, XLSX file, 0.01 MB. © Crown copyright 2021. 2021 Crown This content is distributed under the terms of the Creative Commons Attribution 4.0 International license.

### Presence of Kdn on NTHi LOS does not disrupt IgM antibody and C3 complement deposition.

Human serum bactericidal effects against NTHi are mainly attributed to complement-mediated and antibody-dependent cell cytotoxicity (81, 89, 95, 96). The presence of Sia on NTHi LOS significantly reduces IgM binding to promote resistance against antibody-mediated immune clearance (91, 97). In contrast to IgG, IgM binding is correlated with complement-mediated NTHi killing (98). Compared to Neu5Ac, the presence of Kdn did not alter the deposition of IgG (Fig. S5B and S5C). However, binding of human IgM on bacterial Kdn-LOS was similar to that seen with unsialylated bacteria and significantly higher than observed on Neu5Ac-coated bacteria (Fig. 8A, Fig. S5D). Several bacteria, including NTHi, have evolved efficient mechanisms to inhibit complement pathway activation by interacting with complement inhibitors like factor H (FH) or by binding complement component C3 in a manner that prevents its activation (99–101). For example, NTHi bacteria interact with domains 6 and 7 of FH to inhibit complement activation (81, 96). While the presence of Kdn or Neu5Ac did not alter binding of FH domains 6 and 7 (Fig. S5E and S5F), we detected increased amounts of the C3c fragment of complement component C3 (a measure of C3b and iC3b) on NTHi grown in the presence of Kdn relative to Neu5Ac-treated bacteria (Fig. 8B, Fig. S5G). C3 is a key regulator of innate immunity (102), and all complement pathways converge at the level of C3 deposition, resulting in complement activation that leads to assembly of the membrane attack complex on bacteria. Our data suggest that in contrast to inhibition of IgM deposition and complement activation in the presence of Neu5Ac, the lack of inhibition of C3 fragment deposition and IgM binding to NTHi 2019 surface, grown in the presence of Kdn, keeps them susceptible to serum killing.

### Kdn-glycan epitopes do not interact with Sia-binding immunoglobulin superfamily lectins (Siglecs).

Siglecs are important immunomodulators in vertebrates, including humans (103). NTHi interacts with Siglec-9, an immunoinhibitory receptor on account of its immunoreceptor tyrosine-based inhibitory motif. Engaging immunoinhibitory receptors enables bacteria to escape innate immune recognition. Siglec-9 polymorphisms that are less effective at dampening immune responses are associated with exacerbations of respiratory infections and development of emphysema (104, 105). NTHi also binds paired Siglec-5 (104) and Siglec-14 (106), and polymorphism of Siglec-14 in humans affects inflammatory responses in chronic obstructive pulmonary disease (104). Siglec-5 and -14 have similar extracellular ligand receptors but different cytosolic domains to modulate inhibition and activation, respectively, of the host innate immune response (107). To determine the effect of Kdn on the interaction of Siglecs and sialoglycans, we performed a sialoglycan binding assay with purified human Siglec-5, -9, and -14 (Fig. 8C). Recombinant Siglec proteins were allowed to interact with Sia-terminating glycans on the microarray, and relative binding of each Siglec with Neu5Ac- or Kdn-epitopes was determined (File S3). Siglec-9 showed a diverse range of binding with different Neu5Ac-containing glycans and minimal binding with Kdn-epitopes except for glycans with an underlying lactose residue (marked with asterisks in Fig. 8C). Similar results were observed for both Siglec-5 and -14. While outer membrane proteins also contribute to Siglec-5 and -14 binding, NTHi interaction with Siglec-9 is solely attributed to Neu5Ac (104). We therefore confirmed the binding result of the microarray by measuring binding of recombinant Siglec-9−Fc fusion proteins to NTHi by flow cytometry. While Neu5Ac enhanced binding of Siglec-9−Fc, the presence of Kdn did not alter binding compared to unsialylated bacteria (Fig. 8D). Since Siglec-9 showed high binding only with lactose underlying Kdn-glycans on the microarray (Fig. 8C), we speculate that the lack of Siglec-9 binding to Kdn-coated NTHi may either be due to the scarcity of Kdn-lactose-epitopes and/or low affinity of Kdn for Siglec-9.

### Human-like *Cmah*^−/−^ mice have higher levels of free Neu5Ac in upper airways and are more susceptible to NTHi infection.

**FIG 9:**
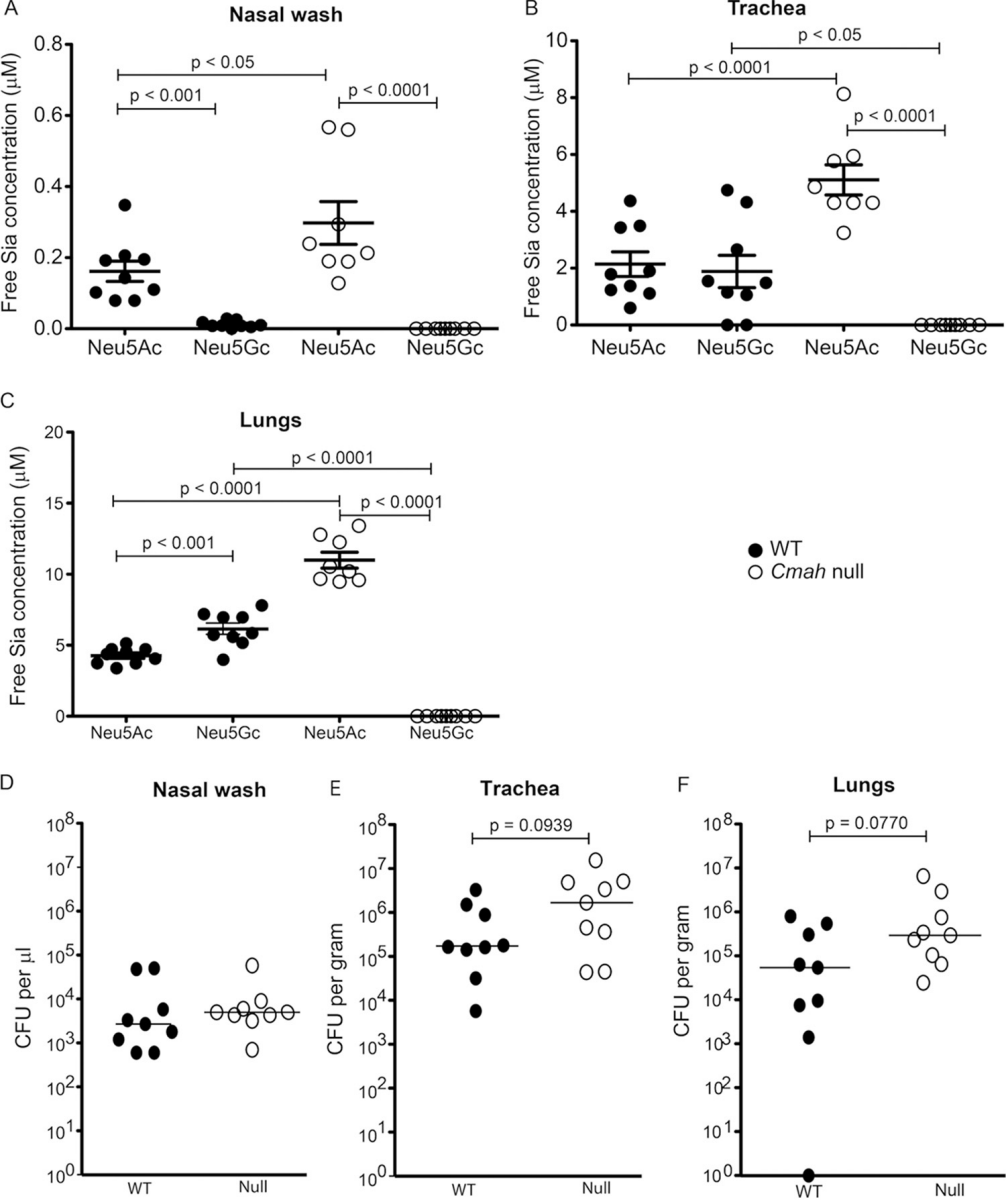
Presence of free sialic acid correlates with *in vivo* infection of NTHi. (A to C) Free sialic acid content of nasal wash (A), trachea (B), and lung (C) of *Cmah* null (open circle) and wild-type (closed circle) mice were determined by HPLC analysis of DMB-derivatized samples of tissue collected 24 h after intranasal infection with NTHi 2019. Statistical significance was determined by one-way ANOVA and Tukey’s multiple comparison test. (D to F) Bacterial load postinfection was determined by the CFU counts of the bacteria from nasal wash (D), trachea (E) and lung (F) homogenates (same samples as used for free sialic acid analysis) that were plated on *Haemophilus* isolation agar. Data represent the results from two independent experiments, with each symbol representing the value for an individual mouse (*n* = 9). Statistical significance was determined using the nonparametric Mann-Whitney test. Adjusted *P* values are shown in the figure.

The importance of Sia in NTHi pathogenesis is well established *in vitro* and *in vivo* (108). The only known natural host of NTHi, humans are unusual compared to most mammals (including chinchilla and mice that have been used to study NTHi pathogenesis *in vivo*) because of their predominantly Neu5Ac-rich sialoglycoconjugates. To address this difference, we infected human-like *Cmah^−/−^* mice with NTHi (109). *Cmah^−/−^* mice have the same exon deletion as humans that inactivates the CMP-Neu5Ac hydroxylase (CMAH) enzyme, resulting in a Neu5Ac-rich sialome (110). Neu5Gc-free *Cmah^−/−^* mice have been utilized to study uniquely human infections and pathophysiology (109, 111, 112). Unlike WT mice that express both Neu5Ac and Neu5Gc, *Cmah^−/−^* mice contained only Neu5Ac in their nasal cavity, trachea, and lungs (Fig. 9A to C). Moreover, free Neu5Ac was significantly higher in airways of *Cmah^−/−^* mice compared to WT (Fig. 9A to C). Upon intranasal challenge of WT and *Cmah^−/−^* mice with NTHi in two independent experiments, higher CFU counts of bacteria were recovered from the trachea and lungs of *Cmah^−/−^* mice compared to WT mice 24 h postinfection (Fig. 9E and F). CFU counts recovered from nasal washes of the two groups did not differ (Fig. 9D). Considering that the human host has a primarily Neu5Ac-based sialoglycome, the lack of difference in the nasal cavities of WT and *Cmah^−/−^* mice compared to trachea and lungs further highlights the significance of free Sia in proper understanding of NTHi *in vivo* infections. We did not detect bacteria in the blood of any infected mice (data not shown).

### Kdn administration reduces NTHi infection in *Cmah^−/−^* mice.

**FIG 10:**
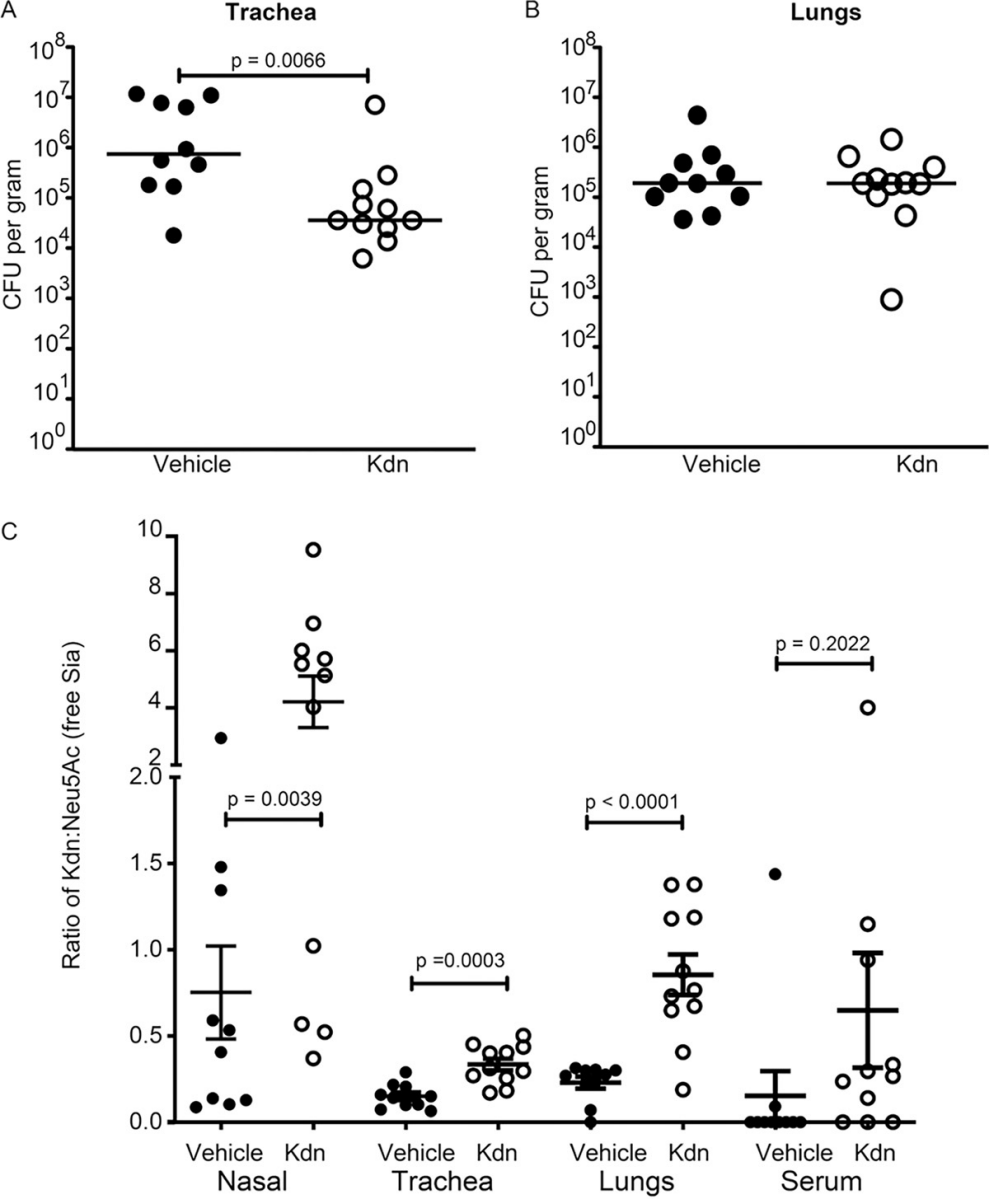
Free Kdn administration reduces NTHi infection in *Cmah* null mice. Following NTHi 2019 infection, free Kdn was administered every 6 h intranasally to the *Cmah* null mice. (A and B) Twenty-four hours postinfection, the mice were sacrificed and bacterial load of trachea (A) and lungs (B) homogenates was determined. Control group of infected mice denoted as “vehicle” were administered with HBSS (used as the solvent for Kdn) at the same corresponding time points as the test condition. Statistical significance was determined using the nonparametric Mann-Whitney test. (C) HPLC analysis of the tissue homogenates from the infected mice was performed to determine the abundance of free Kdn relative to Neu5Ac. Statistical significance was determined using the Student’s *t* test. Mean ± SEM are shown. Data represent the results from two independent experiments, and each symbol represents the value for an individual mouse. Adjusted *P* values are shown in the figure.

Since free Kdn impacted different *in vitro* processes associated with NTHi pathogenesis, we infected *Cmah^−/−^* mice with NTHi and 6 h postinfection administered 5 mM free Kdn intranasally every 6 h. We simultaneously treated a control group with the vehicle for the resuspension of Kdn (HBSS) to control for the physical effects of fluid instillation. Six hours after administration of the third Kdn dose (i.e., 24 h postinfection), we sacrificed mice and harvested the trachea and lungs. Kdn-treated mice showed significant reduction in bacterial CFU counts in the trachea (Fig. 10A), although no difference was noticed in the lungs (Fig. 10B). Since excess Sia is mostly excreted out of the body, we assessed persistence of free Kdn in the mice. Tissues were collected 6 h after the final Kdn dose administration and analyzed for the presence of free Kdn relative to Neu5Ac. HPLC analysis revealed that all tissues of the respiratory tract contain significantly larger amounts of free Kdn compared to Neu5Ac (Fig. 10C). Moreover, sera from the mice showed minimal increases of Kdn, suggesting a localized change in Sia concentrations following intranasal Kdn administration.

## DISCUSSION

Here we demonstrate for the first time that humans develop antibodies against Kdn-terminated glycoconjugates early during the first year of life. While the earliest IgG antibodies likely represent transplacental maternal transfer, subsequent natural immunization probably occurs through normal human microbiome. Antigenic determinants of these antibodies closely resemble the sialoglycoconjugate presentation of human cells. These surface sialoglycans represent self-associated molecular patterns (SAMPs) that are important aspects of healthy and disease processes in the human body (113). Commensals present in different host niches such as *Lactobacillus* (114), *Bifidobacterium* (115), and *Bacteroides* (116) utilize Sias for nutrition (117), pathogens like Neisseria gonorrhoeae, Neisseria meningitidis, Escherichia coli K1, Corynebacterium diphtheriae, Streptococcus agalactiae, Streptococcus pneumoniae, and Campylobacterium jejuni have evolved ways to exploit SAMPs by molecular mimicry to promote resistance against immune clearance. Extensive studies on human microbiome have established its pivotal role in modulating immune responses, including antibody generation (118–120). Commensals also support host defense against pathogenic organisms (121, 122). To test a potential bacterial contribution to anti-Kdn antibody generation, we utilized our knowledge of Sia metabolism in NTHi. NTHi Sia-metabolic enzymes have evolved with Neu5Ac as the preferred substrate (71, 87), and we observed a lower efficiency in Kdn surface incorporation. While our current data do not entirely explain the levels of anti-Kdn antibody present in humans, they nevertheless serve as proof of concept that Sia-sequestering bacteria in the microbiome could act as the antigenic source to trigger the generation of anti-Kdn antibodies. Our data of Kdn assimilation by different NTHi strains (Fig. 4 and 5) as well as the promiscuity of bacterial Sia transporters (123) further suggests that other Neu5Ac-utilizing bacteria could potentially also assimilate Kdn from their environment.

Our work is an attempt to manipulate bacteria’s natural propensity for “molecular mimicry” of host sialoglycans, and we used Kdn as a decoy for Neu5Ac-assimilating pathways in NTHi to guide the immune system against the pathogen. Our data present a unique role of Kdn contributing to the reduction of serum and whole blood survival through the increased deposition of complement C3 and IgM antibody. Kdn incorporation could not only present new targets but also potentially uncover the nonself (bacterial) surface epitopes, otherwise masked by Neu5Ac capping, to immune responders, thus corroborating other antimicrobial mechanisms that target non-Neu5Ac structures (23, 91). The presence of Kdn reduces the engagement of Siglec-9, an inhibitory Siglec widely expressed on circulating leukocytes in humans (124) that Neu5Ac-coated microbes exploit to dampen the immune activation (125). In contrast to Neu5Ac that bacteria including NTHi utilize to evade immune clearance, the presence of Kdn could serve to assist the immune system in eliminating potentially pathogenic Neu5Ac-assimilating microbes. This is further evident in the decreased CFU loads in *Cmah^−/−^* mice upon localized free Kdn administration (Fig. 10), which opens the possibility that Kdn could interfere with microbial colonization in a Kdn-rich niche. Free Kdn synthesis is also reported in Bacteroides thetaiotaomicron (126), a predominant human symbiont that colonizes the naive gut around the first year of life (66). While a comprehensive understanding of its effect on the natural human microbiome, including normal commensals, is yet to be ascertained, the potential implication of Kdn administration is also strengthened by the fact that, although predominantly a lower vertebrate Sia, Kdn is naturally present in humans. Interestingly, free Kdn content in fetal blood is about 2.4 times higher than maternal blood, and there is evidence of free Kdn in human ovaries (34). While database mining suggests that Kdn is ancestral compared to Neu5Ac (Fig. 1; see also Files S1 and S2 in the supplemental material), current evidence of Kdn in bacteria is in pathogens of plants (which do not contain any Sia) (30–32).

Altogether, while extensive studies have demonstrated the significance of Sia and sialylation on bacterial physiology and host-pathogen interactions, the research have mostly focused on Neu5Ac and/or Neu5Gc. This study reveals a yet unexplored aspect of Kdn biology and suggests that it plays important roles in bacterial pathogenesis that are still incompletely understood (Table 1). It is possible that Kdn in humans (produced by Kdn synthase) could act as natural decoy against microorganisms that utilize host Neu5Ac. For such organisms, Kdn could also be explored as a possible therapeutic agent.

**TABLE 1:** Comparison between Sias in relation to human, and commensal/pathogenic bacteria^*a*^

Characteristic	*N*-Acetylneuraminic acid (Neu5Ac)	*N*-Glycolylneuraminic acid (Neu5Gc)	Ketodeoxynonulosonic acid (Kdn)
C-5 structure	*N*-Acetyl	*N*-Glycolyl	Hydroxyl
Occurrence	All Deuterostomes, few Eubacteria, Archaea	Most mammals (not humans)	All vertebrates, some bacteria, algae
Biosynthetic pathway	Condensation of ManNAc-6-P and PEP	CMAH converts CMP-Neu5Ac into CMP-Neu5Gc (only in Deuterostomes)	Condensation of Man-6-P and PEP
Presence in humans	Major Sia, free or glycoconjugate-bound	Glycoconjugate-bound	Free (except in some cancerous tissue?)
		Derived from food	
			
Role in human immunity	Self-associated molecular patterns (SAMPs)	Xeno-autoantigen	Possible autoantigen?
Human Sia-epitope recognizing Abs	Rare	Yes	Yes (this study)
Synthesis by bacteria	Yes (condensation of ManNAc and PEP)	No	Yes (condensation of Man-6-P and PEP)
Presence in bacterial glycans	Yes	Yes, derived from vertebrate host or food	Yes (widespread)
Role in bacteria	Nutrition	Not applicable?	Currently unknown
	Structural roles		Structural roles
Role in bacterium-host interaction	“Molecular mimicry” to evade immune response	Interaction with antibodies?	Engagement of immune response (this study)
Susceptibility to bacterial, viral sialidases	Yes	Yes	Resistant

a Based on multiple references mentioned in the text. Man-6-P, mannose-6-phosphate; PEP, phosphoenolpyruvate.

## MATERIALS AND METHODS

### Bacterial strains and growth conditions.

NTHi human clinical isolate 2019 (NTHi 2019) from chronic obstructive pulmonary disease patient (70, 127), its transporter mutant 2019Δ*siaT* (85), and sialic acid lyase mutant 2019Δ*nanA* (85) were generous gifts from Michael Apicella (University of Iowa). NTHi strains, Rd, 375, 486, 1003, PittGG, and R2846 were generously provided by Richard Moxon (Oxford University, UK), Stephen Pelton (Boston Medical Center), and Brian J. Akerley (University of Mississippi Medical Center) (77–81). NTHi was grown overnight from the frozen stock in chocolate II agar plate (BD BBL) at 37°C for 16 to 18 h in the presence of 5% (vol/vol) CO_2_. The sialic acid-free chemically defined medium used for bacterial growth was as described previously (85, 128). Prior to the bacterial growth, the medium was supplemented with 1 μg/ml protoporphyrin IX (Sigma-Aldrich), 1 μg/ml l-histidine (Sigma-Aldrich), and 10 μg/ml β-NAD (NAD) (Sigma-Aldrich). For the mouse infections, the bacteria were incubated in hemin (Sigma-Aldrich) and NAD-supplemented brain heart infusion (BHI) medium (BD Biosciences) following overnight culture as described above.

### Sialic acid feeding of bacteria.

NTHi was grown on chocolate II agar (84) followed by incubation in the defined media as described above. To eliminate phase variability of the LOS genes, fresh bacterial cultures from frozen stocks were initiated prior to each experiment, and the cultures were never passaged between experiments. Briefly, the day before the experiment, a fresh bacterial culture was started from the frozen glycerol stock (maintained at –80°C) onto BD BBL chocolate II agar, grown overnight (16 to 18 h) at 37°C with 5% CO_2_. On the day of the experiment (i.e., after overnight growth), four or five discrete colonies were incubated in broth for a starter culture. Following 2 to 3 h of growth at 37°C, 200 rpm, early log phase culture (optical density at 600 nm [OD_600_] = 0.075) was further incubated in the presence or absence of 100 µM Sia-Neu5Ac (Nacalai USA, Inc.), Kdn (Cayman Chemical), unless mentioned otherwise, for 4 to 5 h at 200 rpm in 37°C until an OD_600_ of 0.40 was reached. These cultures were used in assays on that day. Surface sialylation of the bacteria used for each assay was confirmed using ECA lectin binding assay. The sialic acid stocks were prepared in phosphate-buffered saline (PBS), pH neutralized, filtered, and stored in aliquots at –20°C until used. Prior to the addition of Neu5Ac or Kdn, the absence of Sia in the chemically defined media was confirmed by HPLC analysis of the DMB-derivatized media.

### Staining with plant lectins and 3F11 antibody.

NTHi grown in the presence or absence of sialic acid was used for lectin staining. Following Sia feeding, the bacterial culture (OD_600_ = 0.1) was spun at 10,000 relative centrifugal force (rcf) for 5 min and washed with HBSS containing CaCl_2_/MgCl_2_. The bacteria were incubated with biotinylated lectins (from Vector Laboratories)—Erythrina cristagalli lectin (ECA) and Maackia amurensis lectin I (MAL I)—at 37°C for 30 min. For 3F11 antibody (generous gift from Michael Apicella, University of Iowa) staining, the bacteria were incubated at room temperature for 30 min. The lectins were used at the following concentration in HBSS with 0.1% BSA-ECA at 1:500 (stock, 5 mg/ml), MAL I at 1:500 (stock, 2 mg/ml) and 3F11 at 1:50 dilution. The binding was detected with either streptavidin conjugated with Cy5 (Jackson ImmunoResearch Laboratories) (1:1,000) or anti-mouse antibody conjugated with fluorescein isothiocyanate (FITC) (Invitrogen) (1:1,000) and visualized using a BD FACSCalibur flow cytometer. The data were analyzed using FlowJo software.

### LOS purification.

All NTHi strains were grown as mentioned above and incubated for 8 h (or overnight) in the presence or absence of sialic acid. Following incubation, the bacteria were washed in PBS and lysed by sonication and freeze-thawing. The cell lysates were incubated with Benzonase nuclease (Millipore Sigma) and proteinase K at 37°C, and LOS was purified using hot phenol-water extraction as described previously (129). The crude LOS was dialyzed against water, lyophilized, and ultracentrifuged at high speed, and the purified LOS was stored at −20°C in PBS.

### HPLC analysis for sialic acid quantification.

HPLC analysis for sialic acid quantification was performed as previously reported (130) with some modifications. Briefly, purified LOS was incubated with 2 N acetic acid for 3 h at 65°C to release the glycosidically bound Sia. Following acid hydrolysis, the samples were derivatized using 1,2-diamino-4, 5-methylenedioxybenzene dihydrochloride (DMB) for 2.5 h at 50°C in the dark. Derivatized samples were next filtered through 10K spin columns (Millipore), and the sialic acid contents were detected with Dionex UltiMate 3000 HPLC system using a Phenomenex Gemini C_18_ column. The excitation and emission signals used for the detection were 373 nm and 448 nm, respectively.

### Detection of free sialic acids.

Homogenates from murine tissues were thawed on ice and spun at 20,000 rpm for 10 min at 4°C to precipitate the cellular debris. Twenty-five microliters of the supernatant was then derivatized using DMB at 4°C for 48 h in the dark (131). At the end of incubation, the derivatized samples were filtered through 10K spin column to eliminate any residual debris and detected using a HPLC machine as described above.

### Bacterial growth curve.

Frozen cultures were struck onto chocolate II agar and incubated overnight at 37°C in 5% CO_2_ (vol/vol). Individual colonies were inoculated in chemically defined media for 2 h at 37°C in 200 rpm. The starter culture was diluted to an optical density (OD_600_) of 0.05, and the bacteria were then grown in the presence or absence of 100 μM sialic acid. To determine growth, the OD_600_ of the bacterial culture was measured every 30 min for 8 h.

### Serum bactericidal assay.

The bactericidal activity of serum was determined as described previously (85). Briefly, mid log phase bacteria, grown in the presence or absence of Sia, were centrifuged at 10,000 rcf for 5 min and washed with prewarmed HBSS. The bacteria were resuspended at OD_600_ of 0.1 in HBSS with 0.1% bovine serum albumin (BSA) and plated on chocolate agar plate following serial dilution (*t* = 0). To determine the sensitivity against normal human serum, 10 μl of the resuspended bacteria was treated with 10% pooled normal human sera (NHS) (Complement Technology) and incubated at 37°C, shaking with 200 rpm for 30 min. Following incubation, the bacteria were serially diluted and plated on chocolate agar (*t* = 30). Resistance to the serum killing was calculated by determining the viable bacterial count at *t* = 30 relative to *t* = 0. The assay was repeated thrice, and for each individual assay, the appropriate sialylation of the bacteria was determined by ECA lectin binding as described above.

### Whole blood killing assay.

For this study, venous blood was collected from healthy donors following informed consent in accordance with the guidelines issued by the Institutional Review Board (IRB 170921), University of California, San Diego (UCSD). The whole blood killing assay was done based on reference 94 with the following modifications. The Sia-fed or unfed bacteria were grown to mid log phase, washed, and resuspended to an OD_600_ of 0.1 in HBSS with 0.1% BSA. The resuspended bacteria were serially diluted and plated on chocolate agar (*t* = 0). To determine the effect of whole blood on survival, the bacteria were diluted 1:10 in freshly collected human blood and incubated at 37°C for 30 min with gentle rotation. Following incubation, the bacteria were serially diluted and grown on chocolate agar (*t* = 30). The percentage of whole blood killing was calculated as the serum bactericidal assay stated above. The assay was repeated with multiple individual blood samples, which were freshly collected in hirudin anticoagulant-containing tubes (Sarstedt).

### Purification of recombinant, soluble Fc-chimeric Siglec proteins.

Siglec proteins were purified as described previously (132). Briefly, culture supernatant from transiently transfected HEK293 cells was collected and incubated with Sepharose protein A Column (GE Healthcare Life Sciences) at 4°C for purification of the soluble Fc-chimeric proteins. After washing with Tris-buffered saline and desialylation with Arthrobacter ureafaciens (Sigma) sialidase to eliminate endogenous sialylation, the Siglec-Fc proteins were eluted with 0.1 M glycine-HCl (pH 3.0) and immediately neutralized using Tris-HCl. The stability of the purified proteins was determined by sodium dodecyl sulfate-polyacrylamide gel electrophoresis (SDS-PAGE).

### Flow cytometry for C3c, IgM, IgG factor H domain 6 and 7 deposition and Siglec-9−Fc binding.

Experiments were performed based on methods described previously (133). Briefly, mid log phase bacteria grown in the presence or absence of Sia were washed in HBSS containing Ca/Mg and then treated with 10% NHS for 5 min at 37°C and 200 rpm. For IgG and IgM deposition, NHS was heat inactivated by incubation at 56°C for 30 min and 200 rpm shaking. Heat-inactivated NHS-treated bacteria were incubated with R-phycoerythrin- or FITC-conjugated antibodies against human IgG and IgM (both Sigma-Aldrich), respectively. For complement component, C3c was probed with FITC-conjugated anti-human C3c (Bio-Rad). For factor H 6/7 binding, the bacteria were incubated with the purified FH6/7 (mouse IgG) supernatant (134) for 30 min and visualized using allophycocyanin (APC)-conjugated anti-mouse IgG. Siglec-9−Fc binding was determined by incubating the bacteria with 300 ng of purified protein for 2 h at 37°C. The visualization was done using Alexa Fluor 488-conjugated secondary antibody against human Fc. All the data were collected using BD FACSCalibur flow cytometer, and the data were analyzed using FlowJo software.

### Animal care.

All animal experiments were conducted under veterinary supervision and approved by the University of California, San Diego Institutional Animal Care and Use Committee (IACUC). Eight- to 10-week-old male and female C57BL/6 and *Cmah^−/−^* (109) mice were bred and maintained in a specific-pathogen-free UCSD animal facility to eliminate any variability introduced by microbiome differences between breeding facilities. Mice were allowed to eat and drink *ad libitum*. All efforts were made to minimize suffering of animals employed in this study.

### *In vivo* intranasal infection of mice.

For intranasal infection of mice, NTHi 2019 was grown as described above and washed with prewarmed HBSS. Eight- to 10-week-old male and female mice were anesthetized with isoflurane and infected intranasally with 4 × 10^8^ to 5 × 10^8^ CFU in 25 µl HBSS. Twenty-four hours postinfection, mice were humanely euthanized via CO_2_ asphyxiation. Trachea and lungs were removed and homogenized in HBSS using a MagNa lyser (Roche). Organ lysates were serially diluted and plated for bacterial CFU enumeration on selective chocolate agar (Remel) incubated at 37°C with CO_2_ for 24 h.

### Sialoglycan microarray.

Chemoenzymatically synthesized sialoglycans were quantitated utilizing DMB-HPLC analysis, and glycans were dissolved in 300 mM sodium phosphate buffer (pH 8.4) to the final concentration of 100 µM. NHS-functionalized glass slides (PolyAn 3D-NHS slides from Automate Scientific) were printed with the sialoglycans using a ArrayIt SpotBot Extreme instrument. The method described previously (56) was used with the following modifications. Each glycan was printed in quadruplets. The temperature (20°C) and humidity (70%) inside the ArrayIt printing chamber was thoroughly maintained during the printing process. The slides were left for drying for an additional 8 h. Printed glycan microarray slides were blocked with prewarmed 0.05 M ethanolamine solution (in 0.1 M Tris-HCl [pH 9.0]), washed with warm Milli-Q water, dried, and then fitted in a multiwell microarray chamber (Grace Bio-Labs) to divide into 16 subarrays. Each subarray well was treated with 200 µl of ovalbumin (1% [wt/vol]) dissolved in freshly prepared PBS buffer (pH 7.4) for 1 h at ambient temperature in a humid chamber with gentle shaking. Subsequently, 200 µl of serum solution (1:250 dilution for IgG and 1:50 dilution for IgM detection) was added to the subarray. After incubating for 2 h at room temperature with gentle shaking, the slides were extensively washed and treated with fluorophore-conjugated secondary antibodies. Goat anti-human IgG and IgM conjugated to Cy3 and Cy5, respectively (Jackson ImmunoResearch) were used. Finally, the microarray slides were dried and scanned with a Genepix 4000B (Molecular Devices Corp., Union City, CA) microarray scanner (at 532/635 nm) and data analysis using the Genepix Pro 7.3 analysis software and plotted in Microsoft Excel.

### Human serum samples.

The human serum samples from mothers and children at birth and 3, 6, and 12 months old used in the sialoglycan microarray experiments were the same as previously published (48). The sera were collected previously in accordance with the approved protocols of University of La Frontera, Temuco, Chile, Institutional Review Board and Regional Ethical Committee of the Chilean National Health Service for the Araucania Region. On the basis of the dietary history, the mothers during their pregnancy and in the first months during breast feeding routinely consumed plant products like apples, peaches, tomatoes, avocados, lettuce, cranberries, blueberries, broccoli, eggplant—all of which are rich sources of dietary mannose. The children fed only on mother’s milk and did not receive any mammalian meat during the first 3 months. All the samples were completely anonymized and deidentified prior to receiving the samples at UCSD for the studies.

Human blood for all the assays involving the sera and whole blood were collected from healthy adult volunteers with informed consent in accordance with the approved institutional protocols of UCSD human research protections program (IRB 170921). The commercially available pooled normal human IVIG used in the glycan binding, serum killing, and immune factor interaction studies were bought from Complement Technology and used with appropriate safety precautions.

### Statistical analysis.

Statistical significance was determined with GraphPad Prism version 8 software mainly using one-way analysis of variance (ANOVA) with Tukey multiple comparison test, nonparametric Mann-Whitney test, or two-tailed paired Student *t* test as stated in the figure legends, unless mentioned otherwise. The statistical analysis of the mouse infection data (Fig. 9D to F and Fig. 10A and B) was done using nonparametric Mann-Whitney test.
