# Supplementary material for: Exploring the Impact of Ketodeoxynonulosonic Acid in Host-Pathogen Interactions Using Uptake and Surface Display by Nontypeable Haemophilus influenzae
Source: mBio. 2021 Jan 19;12(1):e03226-20. doi: 10.1128/mBio.03226-20 (PMC7845648; doi:10.1128/mBio.03226-20)
Supplement: TABLE S1 [file mBio.03226-20-st001.docx]

**Supplemental Table S1: Comparison between Kdn and Kdo***

(*Based on multiple references mentioned in the text)

|  | **Kdn** | **Kdo** |
| --- | --- | --- |
| **Backbone** | 9-carbon | 8-carbon |
| **Occurrence** | All vertebrates, some bacteria, algae | All Gram-negative bacteria, some algae, most higher plants |
| **Structure** |  |  |
| **Glycosidic linkage** | α | α and β |
| **Biosynthetic pathway** | Condensation of Mannose-6-P and PEP | Condensation of Arabinose-5-P and PEP |
| **Presence in human glycome** | Yes | No |
| **Synthesis by bacteria** | Yes, also assimilated from environment (this study) | Yes, Conserved pathway in Gram-negative bacteria |
| **Presence of recognizing antibody in human** | Yes  (this study) | Unknown |
| **Functionality in bacteria** | Structural (?)  Immune evasion (this study) | Component of some capsular polysaccharides and core component of lipopolysaccharides |
