# Supplementary figures and images for: Exploring the Impact of Ketodeoxynonulosonic Acid in Host-Pathogen Interactions Using Uptake and Surface Display by Nontypeable Haemophilus influenzae
Source: mBio. 2021 Jan 19;12(1):e03226-20. doi: 10.1128/mBio.03226-20 (PMC7845648; doi:10.1128/mBio.03226-20)

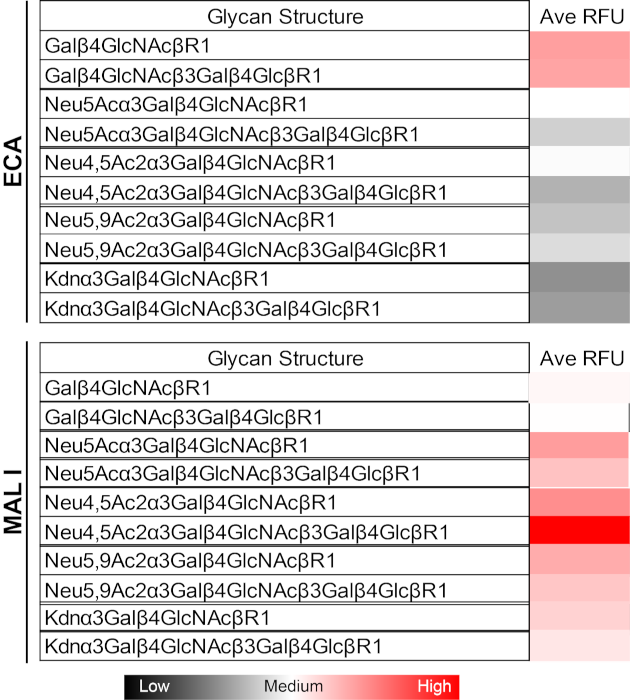

Supplement: FIG S1 [file mBio.03226-20-sf001.tif]

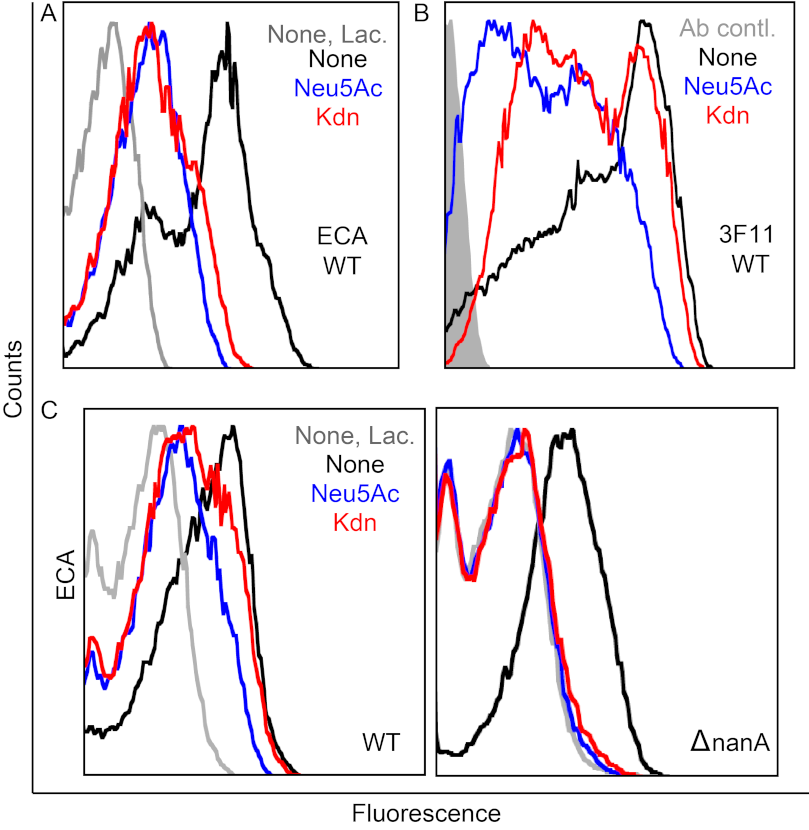

Supplement: FIG S2 [file mBio.03226-20-sf002.tif]

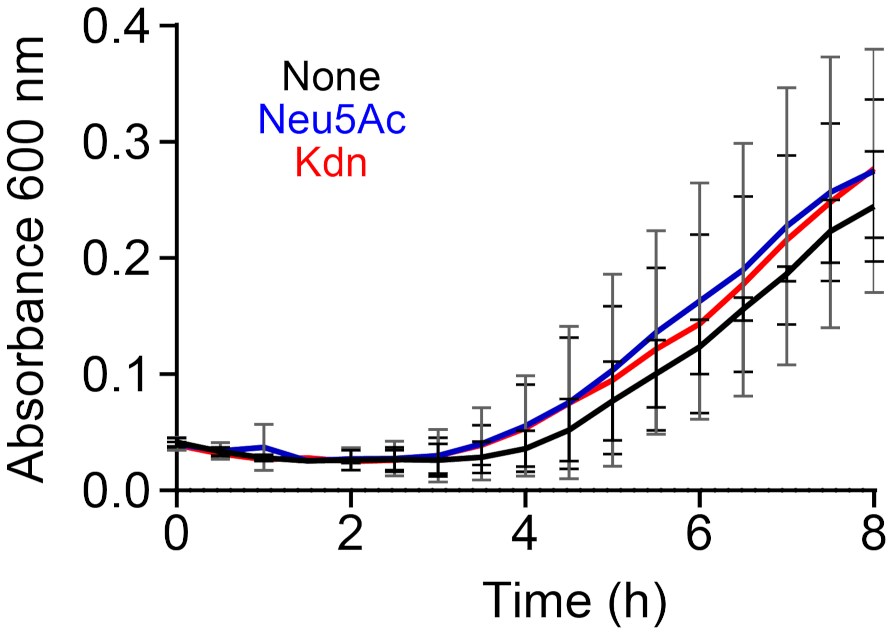

Supplement: FIG S3 [file mBio.03226-20-sf003.tif]

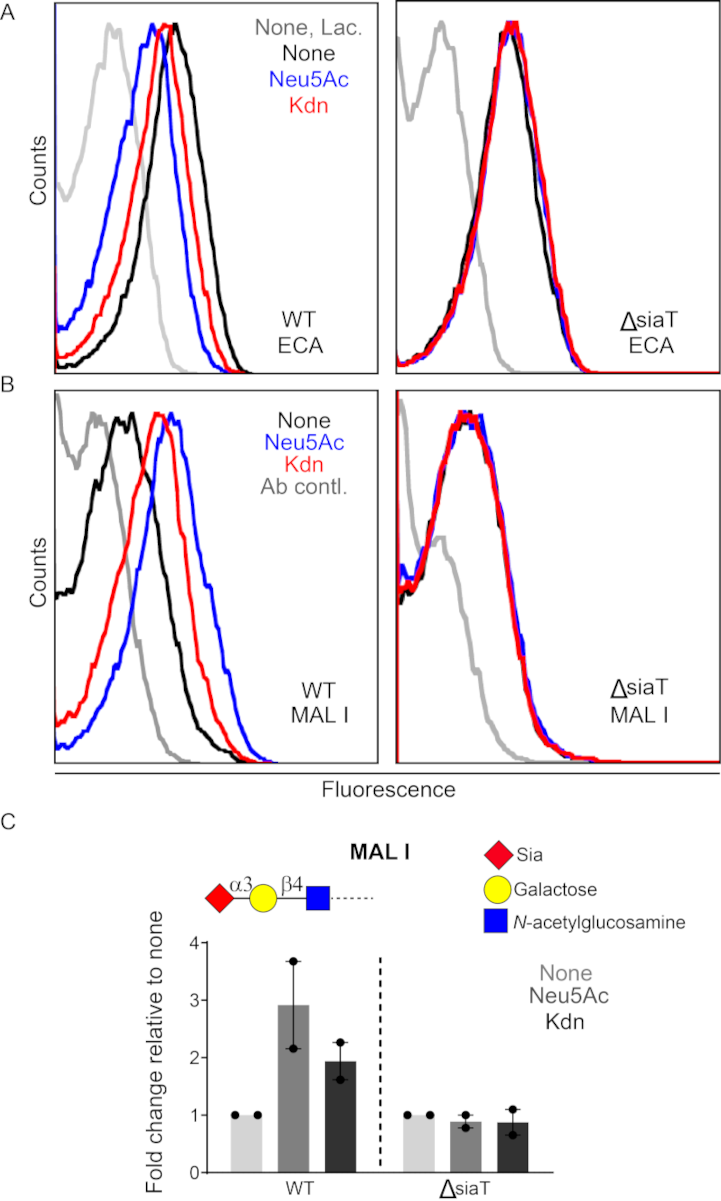

Supplement: FIG S4 [file mBio.03226-20-sf004.tif]

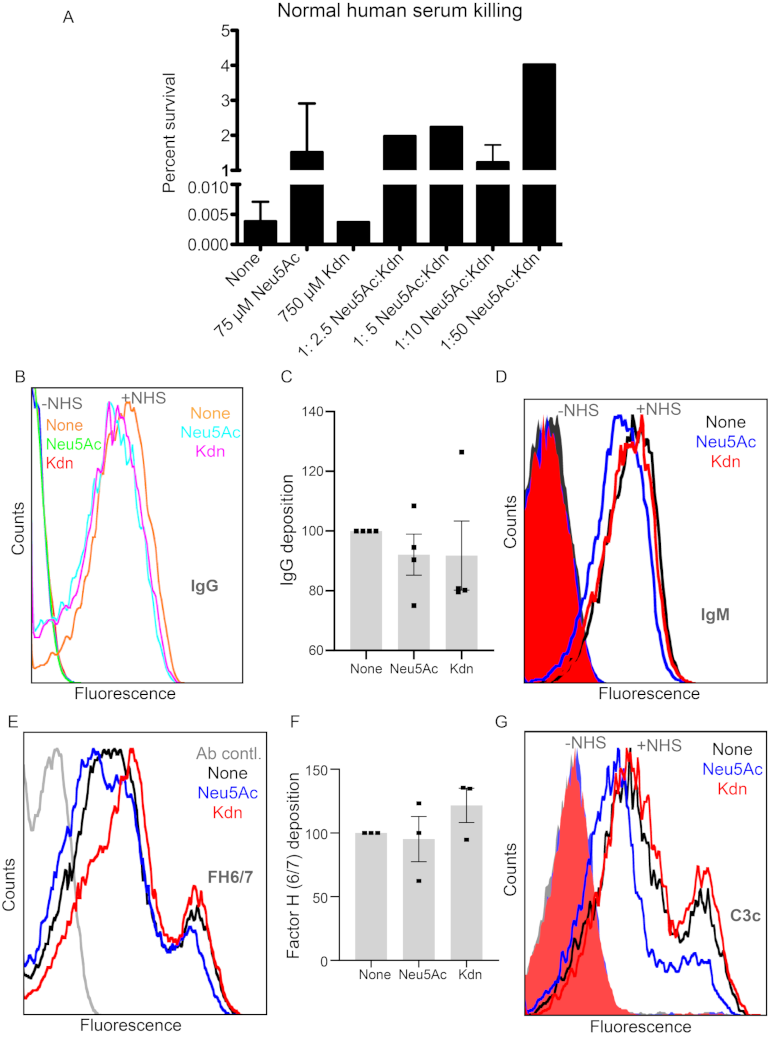

Supplement: FIG S5 [file mBio.03226-20-sf005.tif]
